# Linking Abundance and Activity of Ammonia‐Oxidising Bacteria and Archaea in an Agriculturally Impacted First‐Order Stream

**DOI:** 10.1111/1462-2920.70369

**Published:** 2026-07-13

**Authors:** Zhe Wang, Anna Störiko, Aileen Jung, Daniel Straub, Olaf A. Cirpka, Holger Pagel, Tillmann Lueders

**Affiliations:** ^1^ Chair of Ecological Microbiology, Bayreuth Center of Ecology and Environmental Research (BayCEER), University of Bayreuth Bayreuth Germany; ^2^ Department of Earth and Planetary Sciences ETH Zürich Zürich Switzerland; ^3^ Department of Geosciences University of Tübingen Tübingen Germany; ^4^ Department of Water Management Delft University of Technology Delft the Netherlands; ^5^ Quantitative Biology Center University of Tübingen Tübingen Germany; ^6^ Department of Biogeophysics University of Hohenheim Stuttgart Germany; ^7^ Institute of Bio‐ and Geosciences IBG‐3: Agrosphere Forschungszentrum Jülich GmbH Jülich Germany; ^8^ Institute of Crop Science and Resource Conservation University of Bonn Bonn Germany

**Keywords:** 16S rRNA sequencing, functional genes, microbial abundance, nitrifiers, reaction modelling, reactive nitrogen, streambed sediments

## Abstract

Lower‐order streams in agricultural landscapes receive major anthropogenic nitrogen inputs. Streambed sediments host diverse microbial communities that can influence nitrogen (N) fluxes and water chemistry. Both bacterial and archaeal ammonia oxidizers inhabit streambeds, but their respective contributions to nitrification are often unresolved. We investigated a first‐order stream in southern Germany to assess the contribution of distinct ammonia‐oxidising populations to streambed nitrification. We combined in situ geochemical data, 16S rRNA and functional‐gene amplicon sequencing, quantitative PCR and microcosm incubations with selective chemical inhibitors. A process‐based reaction model quantified total nitrification rates and inferred contributions of ammonia‐oxidising archaea (AOA) and bacteria (AOB), while population‐specific kinetic parameters were estimated using Bayesian inference. We found that AOB dominated nitrification and responded more strongly to ammonium inputs than AOA despite being less abundant. Among them, populations of *Nitrosomonas* and *Nitrosospira* spp. were most important. Differences in ammonia‐oxidation rates and ammonia‐oxidising communities between sediment depths and successive stream segments suggest a hydrological influence on streambed nitrification. Our study demonstrates the strength of combining field data, microcosm incubations and modelling to better understand microbial N‐cycling in the environment. It also mandates caution when interpreting functional‐gene abundance as a proxy for in situ reactive potential.

## Graphical abstract

Ammonia‐oxidising archaea (AOA) and bacteria (AOB) coexist in streambed sediments, but their contributions to nitrification remain unresolved. Using field sampling, sequencing, qPCR, microcosm incubations and modelling, we show that AOB dominated nitrification in streambed sediments despite lower abundance, emphasizing the importance of distinguishing abundance from activity in microbial nitrogen cycling.

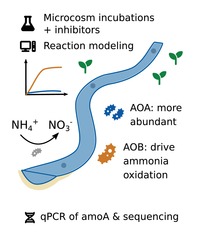

## 1 Introduction

Intensive agriculture causes the input of excess reactive‐nitrogen species into the water cycle, leading to major pollution of streams (David and Gentry 2000; Bouwman et al. 2005; Galloway et al. 2008; Halvorson et al. 2014). Nitrogen reaches first‐ and second‐order streams mainly via surface runoff and groundwater discharge (Grose et al. 2022), substantially affecting the water quality of downstream higher‐order streams and rivers (Peterson et al. 2001; Alexander et al. 2007).

Stream hydrology and microbiology are relevant in controlling nitrogen turnover in streams (Arango and Tank 2008), especially when the buffer capacity of adjacent agricultural soils is limited. Streambed sediments have a significant impact on the transformation of nitrogen species (Newbold 1992; Butturini et al. 2000). Both nitrification and denitrification are considered to be controlled by hyporheic flow and indigenous microbial populations (Krause et al. 2013; Nogaro et al. 2010). Depending on hyporheic flow paths and redox conditions in streambed sediments, the streambed can act as a sink or source of reactive‐nitrogen species (Triska et al. 1993; Storey et al. 2004). The strength of hyporheic exchange is often reduced in agricultural lower‐order streams due to dredging and straightening of the streams to improve drainage and increase crop production (Blann et al. 2009; Hanrahan et al. 2018). Nevertheless, surface water–groundwater exchange has been observed also in such a modified lower‐order streams (Jimenez‐Fernandez et al. 2022; Wang et al. 2022). Gaining and losing conditions in successive segments of the stream have been observed to affect the biogeochemical potential for denitrification in streambeds (Wang et al. 2022). Variable hydrogeological conditions may therefore also impact the distribution and contributions of ammonia‐oxidising microbes. Understanding the controlling factors could be relevant to understand potential secondary nitrate loading in the stream via nitrification upon groundwater discharge (Peterson et al. 2001; Storey et al. 2004; Duff and Triska 2000).

The first step of nitrification, ammonia oxidation, is considered a key process in the nitrogen cycle. It is typically mediated by aerobic autotrophs, either ammonia‐oxidising bacteria (AOB) or ammonia‐oxidising archaea (AOA) (Cardarelli et al. 2020). Typical AOBs are found in the families *Nitrosomonadaceae* (Betaproteobacteria) and *Nitrosococcaceae* (Gammaproteobacteria) (Cardarelli et al. 2020), whereas an important AOA phylum, the Thaumarchaeota, includes members of *Nitrososphaeraceae* and *Nitrosopumilaceae* (Jung et al. 2021). Both AOA and AOB perform ammonia oxidation through ammonia monooxygenase (AMO), a multi‐subunit enzyme (Kuypers et al. 2018). The *amoA* gene encoding the subunit A of AMO is widely used as marker gene to study the diversity and abundance of AOA and AOB populations (Ramanathan et al. 2017; Spasov et al. 2020). The recent discovery of complete ammonia oxidation (comammox) has revealed another important driver of ammonia oxidation in various environments (Van Kessel et al. 2015; Xia et al. 2018). In addition, reduced nitrogen can also be oxidised heterotrophically by chemoorganotrophic bacteria and eukaryotes (e.g., fungi) (Stein 2014; Zhu et al. 2015).

The niche partitioning of prokaryotic ammonia oxidizers and their contributions to nitrification in different ecosystems are still not fully understood. Even though AOB, AOA and comammox bacteria co‐occur in diverse environments, AOB are typically reported as dominant microorganisms under nitrogen‐rich conditions, whereas AOA are assumed to be more important in oligotrophic systems (Zhang et al. 2021; Leininger et al. 2006; Verhamme et al. 2011; Jiang et al. 2014; Yang et al. 2016). Within AOB, the *Nitrosospira* genus is often dominant in terrestrial environments, especially in unfertilised soil and under oxygen‐limited conditions, whereas *Nitrosomonas* phylotypes are more abundant in fertilised and ammonia‐rich environments (Aigle et al. 2019; Norton et al. 2002; Wagner et al. 1996; Xia et al. 2005). Only a few studies have focused on nitrification in agricultural lower‐order streams (Peterson et al. 2001; Arango and Tank 2008; Strauss and Lamberti 2002). Hence the distribution and contributions of distinct ammonia‐oxidising microorganisms to oxidative N‐cycling is not well understood. The relationship between *amoA* gene abundance inferred by qPCR and nitrification rates also remains unclear (Prosser 2012).

To address these issues, our study focuses on three main questions: (1) What are the relative contributions of AOA and AOB to ammonia oxidation in streambed sediments of an agricultural stream? (2) Which ammonia oxidizers are present in these sediments and respond to ammonium pulses? (3) Do the in situ *amoA* gene abundances allow us to predict population‐specific contributions to nitrification? We hypothesised that contributions of AOA and AOB to ammonia oxidation would exhibit spatial patterns (with depth and along the stream) linked to local hydrology and that the high‐nutrient conditions typical of agricultural runoff primarily favour AOB as dominant drivers of nitrification.

We approached these questions by combining microcosm incubations with process‐based modelling. Samples obtained from different stream depths and segments of a well‐characterised first‐order agricultural stream (Jimenez‐Fernandez et al. 2022; Wang et al. 2022) were used to investigate the heterogeneity of nitrification kinetics within the stream. We exposed streambed ammonia oxidizers to a pulse of ammonia, discerning AOA and AOB contributions by applying specific chemical inhibitors. The resulting chemical data as well as AOA and AOB abundances were used to calibrate a process‐based reaction model with Bayesian uncertainty estimation, allowing us to infer population‐specific kinetic parameters and quantify their respective contributions to nitrification.

## 2 Materials and Methods

### 2.1 Site Description and Sampling

The first‐order stream Schönbrunnen (48.32° N and 8.57° E) is a tributary of the second‐order stream Käsbach, situated close to the city of Tübingen, in South‐West Germany. The geology of the catchment is dominated by mud‐ and dolostones of the upper Triassic Erfurt formation and Quaternary loess deposits. The Schönbrunnen has a mean discharge of approximately 1 L/s. The northwestern part of the catchment is extensively developed due to farming and pasture activities. Stream sediments were collected from a 550 m segment of the Schönbrunnen in June 2020 by taking sediment push‐cores using a stainless‐steel piston corer (Eijkelkamp, Giesbeek, Netherlands). After coring, the sediments were placed onto a clean plastic furrow‐shaped tray for depth differentiation. Subsamples from two distinct depths (5 ± 2 cm and 15 ± 2 cm below sediment surface) were collected using a sterile spatula and stored in sterile 50 mL PE tubes (Fisher Scientific GmbH, Schwerte, Germany). Collected samples were then transported to the lab on dry ice (excl. sediment samples that were collected to set up microcosm incubation) and processed within 3 days. Before the collection of sediment at the Schönbrunnen, the studied area did not experience major precipitation or flooding events within a month.

Stream water and adjacent groundwater samples were collected in 1 L glass bottles after the collection of sediment samples. Groundwater was extracted from monitoring wells using a portable peristaltic pump. The water table ranged from 0.5 to 2.0 m below ground level. Water samples were filtered through 0.22 μm Corning Sterile Disposable Filter Systems (Corning Incorporated Life Sciences, Acton, MA, USA) within 48 h. Pore‐water samples were acquired from the sediment using mini piezometers (≤ 2.5 mL/min) (Duff et al. 1998). All filtered samples were kept at 4°C in the dark until further analysis by ion chromatography (Dionex DX 500, Thermo Fisher Scientific, Waltham, MA, USA). Schönbrunnen stream discharge (Q), groundwater table and major water chemistry parameters are listed in Table S1.

### 2.2 Processing of Sediment Samples and Setup of Microcosm Experiments

**FIGURE 1:**
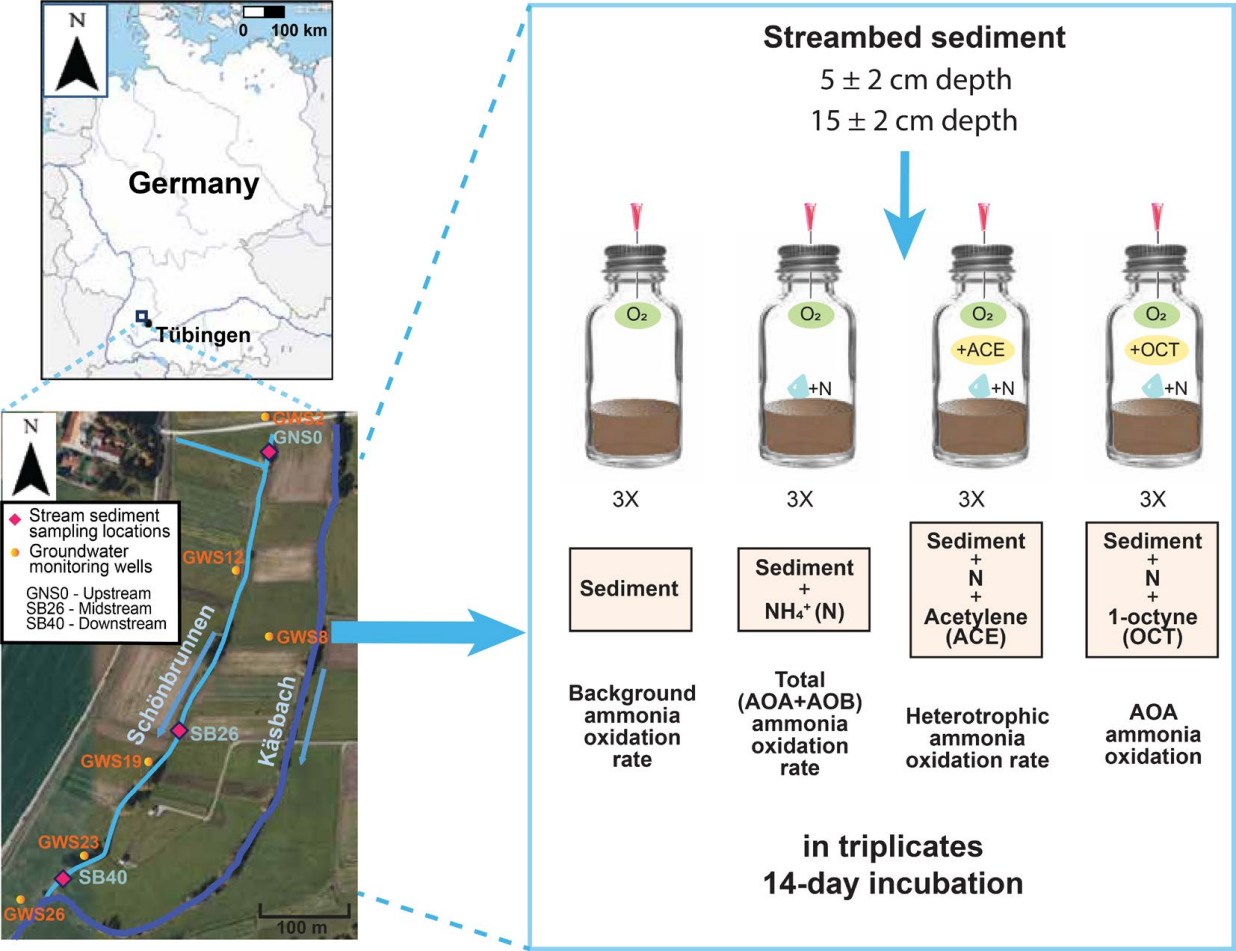
Location of the Schönbrunnen study site and schematic description of the microcosm setup. Ammonia‐oxidation activity in streambed sediments (sampled at 5 and 15 cm depth) obtained from upstream (Up), midstream (Mid) and downstream (Down) sections of the Schönbrunnen was investigated. Sediment microcosms were prepared with three replicates for each treatment: sediment amended with ammonium (Sedi + N), sediment amended with ammonium and acetylene (Sedi + N + ACE), sediment amended with ammonium and 1‐octyne (Sedi + N + OCT) and a control treatment.

Fresh sediments obtained at the Schönbrunnen were preprocessed for microcosm incubation (Figure 1). Samples included streambed sediment obtained at 5 and 15 cm depths from three stream segments: upstream, midstream and downstream. Larger pebbles and debris were manually removed from the samples before transfer to the lab. Within 24 h, the sediment samples were sieved through analytical sieves with successive mesh sizes of 8.0 and 2.0 mm to remove coarser particles and plant material. Sieved sediments from replicate cores were pooled to obtain a homogenised wet sediment slurry. The gravimetric water content of sieved sediment was measured prior to the setup of the microcosm experiments. Sediment slurries were prepared with 10% ± 2.6% (dry weight) sediment inoculum in sterilised simulated stream‐water medium (Table S2). The medium—modified from de la Torre et al. (2008)—was formulated with major ions (NaCl, MgCl_2_, CaCl_2_, KCl and NaHCO_3_) to simulate the in situ neutral to slightly alkaline water chemistry (pH 6.9–7.8) and background buffering capacity of the stream (Jimenez‐Fernandez et al. 2022). The final pH of the slurry was 7.5. Each portion of blended slurry (45 ± 1 g) was subsequently added to sterilised 125 mL MK‐bottles (Müller + Krempel, Bülach, Switzerland), sealed with a rubber top and an aluminium cap. In total, three sets of treatment groups and three sets of control groups were prepared per depth and stream segment (Figure 1).

All treatment groups were supplemented with ammonium to attain a final concentration of 0.5 mM, reflecting the medium‐to‐high range of concentrations observed in the catchment (Table S1). This concentration was chosen to ensure substrate‐saturated conditions at the start of the experiment, thereby allowing for the determination of maximum potential nitrification rates and ensuring that across‐sample comparisons reflected differences in microbial community potential. In addition, this nominal addition was designed to ensure sufficient dissolved substrate availability at the start of the incubation, considering potential rapid cation exchange and adsorption to the sediment matrix (Mackin and Aller 1984). There were three treatment groups (Figure 1):

1. An incubation group amended with ammonium without chemical inhibitors. This was set up for determining total ammonia oxidation from both heterotrophic and autotrophic microbial communities.
2. An incubation group amended with ~6 μM acetylene, to infer the contribution of heterotrophic ammonia oxidation (the activity of both AOA and AOB is supposed to be inhibited by acetylene).
3. An incubation group amended with ~4 μM 1‐octyne, to infer the contribution of AOA‐driven ammonia oxidation (the activity of AOB is supposed to be inhibited by 1‐octyne).

The selection of these concentrations was based on established methodological benchmarks (Taylor et al. 2013; Giguere et al. 2015). Specifically, previous pure‐culture and soil slurry experiments have demonstrated that ~1–5 μM of 1‐octyne irreversibly inactivates bacterial AMO while remaining well below the threshold that would negatively impact archaeal AMO, thereby ensuring group‐specific selectivity. Concurrently, an aqueous concentration of 6 μM acetylene was sufficient for the complete inactivation of AMO across both domains.

The acetylene and 1‐octyne stock solutions were prepared as reported previously (Giguere et al. 2015). In the first control group, a ‘dead’ control was set up to determine abiotic ammonia oxidation. For this, the sediment slurry was treated with three repetitive cycles of autoclaving (121°C, 20 min) and amended with ammonium to a final concentration of 0.5 mM. A second control group contained live slurry without ammonium amendment. The third control group contained mixed stream water from three stream segments, without sediments. These control incubations were further mixed with the same simulated stream‐water medium as used for all slurry incubations and supplemented with 0.5 mM ammonium, to assess ammonia oxidation in stream water. Actual stream water accounted for 60% of the volume; the final composition was similar to all other treatments. All incubations were prepared in triplicates except for the water control group, which was prepared in duplicates. Incubation was performed in the dark at 15°C and the microcosms were agitated on an orbital shaker with a constant speed of ~90 rpm for 14 days, maintaining ambient oxygen conditions until the end of the incubation. Slurry samples for subsequent DNA isolation were taken on Days 0, 5, 10 and 14. Samples taken from Days 0 and 14 were processed for qPCR analyses of 16S rRNA and *amoA* genes.

To collect samples for ammonium measurements, 2 mL of the targeted homogenised slurry sample were centrifuged at 14,000 rpm for 5 mins. The supernatant was further filtered through a 0.22 μm nylon syringe filter (Sigma‐Aldrich, Darmstadt, Germany) and stored at −20°C until further measurement. The sediment pellets were stored at −80°C for subsequent molecular‐biological analyses. The pH of each incubation was between 7.4 and 7.6 throughout the incubation. During the process of sample collection, incubations were opened to ensure ambient oxygen conditions. After the sampling, the microcosms were tightly sealed again. Subsequently, acetylene and 1‐octyne were replenished to reestablish inhibitory concentrations. The determination of ammonium concentrations was performed according to a colorimetric method described previously (Gadkari 1984). Concentrations of nitrite and nitrate were quantified by ion‐exchange chromatography (Metrohm, Herisau, Switzerland) in the Analytical Chemistry Keylab of BayCEER, University of Bayreuth.

### 2.3 Process‐Based Modelling of Nitrification

A process‐based model simulating nitrification in the microcosm experiments was set up to support the interpretation of the data. The model describes ammonia oxidation by AOA and AOB and the associated growth dynamics. Additionally, it accounts for the growth of non‐nitrifying archaea and bacteria and the growth of AOA through processes other than nitrification. Based on the initial assessment of measured dynamics of NH_4_^+^, nitrate (NO_3_^−^) and microbial gene abundances, the modelling approach considered nitrification‐controlled NH_4_^+^‐release from sediments and NH_4_^+^ uptake and incorporation by all biomass pools. While AOA and AOB chemically oxidise ammonia (NH_3_), we formulated our model with respect to ammonium (NH_4_^+^) concentrations, as this was measured in the study. An equilibrium‐speciation calculation considering dissolved ammonia and ammonium and gas‐phase ammonia also showed that > 98% is present as NH_4_^+^ under the conditions of the experiment. At constant pH, the ammonia concentration can be considered proportional to the ammonium concentration due to the quick equilibration between the two species.

The oxidation of NH_4_^+^ and the corresponding growth of AOA and AOB through this process are described by Monod‐type rate laws. The oxidation rate of NH_4_^+^ by group i (relating to either AOA or AOB) is given by:

(1) $$r_{\mathrm{NH}₄⁺}^{i}=\nu_{\max}^{i}\frac{c_{\mathrm{NH}₄⁺}}{c_{\mathrm{NH}₄⁺}+K_{\mathrm{NH}₄⁺}^{i}}f_{\text{inhib}}^{i}B_{i}$$

where $\nu_{\max}^{i}$ [mol cell^−1^ s^−1^] is the maximum cell‐specific NH_4_^+^‐oxidation rate, $c_{\mathrm{NH}₄⁺}$ [mol L^−1^] and $K_{\mathrm{NH}₄⁺}^{i}$ [mol L^−1^] are the NH_4_^+^‐concentration and half‐saturation constant and $B_{i}$ [cells L^−1^] is the cell density of group i. To account for the inhibition of nitrification rates via 1‐octyne or acetylene, the rate contains the factor $f_{\text{inhib}}^{i}$ which ranges between 0 (complete inhibition) and 1 (no inhibition). We assume that acetylene leads to complete inhibition of AOA and AOB. For 1‐octyne, we estimate the respective values of $f_{\text{inhib}}^{i}$ for AOA and AOB from the data (see Section 4.3 for a discussion of this approach). The growth rate of AOA and AOB through NH_4_^+^‐oxidation and, hence, nitrification, is proportional to the NH_4_^+^‐oxidation rate given by:

(2) $$r_{\text{growth},\mathrm{NH}₄⁺}^{i}=Y_{i}r_{\mathrm{NH}₄⁺}^{i}$$

with the growth yield $Y_{i}$ [cells mol^−1^].

Lacking accumulation of nitrite (NO_2_^−^) during the experiment indicated that NH_4_^+^‐oxidation was the rate‐limiting step. Therefore, production and consumption of NO_2_^−^ are not explicitly considered and the production of NO_3_^−^ is directly derived from the sum of the nitrification rates:

(3) $$\frac{dc_{\mathrm{NO}₃⁻}}{\mathrm{dt}}=\sum_{i}r_{\mathrm{NH}₄⁺}^{i}$$

The NO_3_^−^ amount produced in the treatments without inhibitor and with the addition of 1‐octyne exceeded the NH_4_^+^ amount initially added to the microcosms. However, NH_4_^+^‐concentrations remained constant when nitrification was inhibited entirely by acetylene. Based on this initial data evaluation, the model was formulated assuming that organic N was additionally released from the sediments and that this additional N‐release depends on active nitrification. Therefore, the NH_4_^+^‐release from the sediment changes proportionally to the total nitrification rate:

(4) $$r_{\text{release}}^{\mathrm{NH}₄⁺}=\alpha_{\text{release}}\sum_{i}r_{\mathrm{NH}₄⁺}^{i}$$

with the dimensionless proportionality factor $\alpha_{\text{release}}$.

Preliminary data analysis indicated that growth of AOA also occurred in the absence of nitrate production. We therefore tested the inclusion of an alternative growth process that is independent from ammonium oxidation:

(5) $$r_{\mathrm{alt}}^{\mathrm{AOA}}=\mu_{\mathrm{alt}}^{\mathrm{AOA}}B_{\mathrm{AOA}}$$

where $\mu_{\mathrm{alt}}^{\mathrm{AOA}}$ [s^−1^] is the specific growth rate of AOA from the alternative growth process.

Both, AOA and AOB pools decay according to a first‐order rate law

(6) $$r_{\mathrm{dec}}^{i}=k_{\mathrm{dec}}^{i}B_{i}$$

with the decay coefficient $k_{\mathrm{dec}}^{i}$ [s^−1^].

The dynamics of AOA and AOB are given by summing up all growth and decay terms:

(7) $$\frac{dB_{\mathrm{AOA}}}{\mathrm{dt}}=r_{\text{growth},\mathrm{NH}₄⁺}^{\mathrm{AOA}}+r_{\mathrm{alt}}^{\mathrm{AOA}}-r_{\mathrm{dec}}^{\mathrm{AOA}}$$

(8) $$\frac{dB_{\mathrm{AOB}}}{\mathrm{dt}}=r_{\text{growth},\mathrm{NH}₄⁺}^{\mathrm{AOB}}-r_{\mathrm{dec}}^{\mathrm{AOB}}$$

The growth of microorganisms requires an N‐source for biomass production. We assume that microorganisms use NH_4_^+^ as their N‐source. The consumption rate of NH_4_^+^ for incorporation into biomass is then given by:

(9) $$r_{\text{incorporation}}=\sum_{i}\beta_{i}r_{\text{growth}}^{i}$$

where the index i stands for archaea or bacteria, $\beta_{i}$ is the number of moles of nitrogen per cell and $r_{\text{growth}}^{i}$ is the total growth rate of all archaea/bacteria, including nitrifiers and non‐nitrifiers (Equations 12 and 13). The dynamics of NH_4_^+^‐concentrations are then given by:

(10) $$\frac{dc_{\mathrm{NH}₄⁺}}{\mathrm{dt}}=r_{\text{release}}^{\mathrm{NH}₄⁺}-\sum_{i}r_{\mathrm{NH}₄⁺}^{i}-r_{\text{incorporation}}$$

Our experimental data did not provide information on the metabolism of non‐nitrifying organisms, but only on the total abundances of archaea and bacteria. The model therefore considers two groups (archaea and bacteria) of non‐nitrifiers whose biomass is given by:

(11) $$\frac{dX_{i}}{\mathrm{dt}}=r_{X}^{i},$$

with a constant growth rate $r_{X}^{i}$ [cells L^−1^ s^−1^].

The total growth rates for all archaea and bacteria are given by:

(12) $$r_{\text{growth}}^{\text{archaea}}=r_{\text{growth},{\mathrm{NH}}_{4}^{+}}^{\mathrm{AOA}}+r_{\mathrm{alt}}^{\mathrm{AOA}}+r_{X}^{\text{archaea}}$$

(13) $$r_{\text{growth}}^{\text{bacteria}}=r_{\text{growth},\;{\;\mathrm{NH}}_{4}^{+}}^{\mathrm{AOB}}+r_{X}^{\text{bacteria}}$$

Abundances of *amoA* genes were calculated from the simulated biomass of AOA and AOB assuming that a single cell contains 1, respectively, 3 *amoA* gene copies (Lagostina et al. 2015). Abundances of 16S rRNA genes were computed for archaea and bacteria separately using the simulated summed biomass of nitrifying and non‐nitrifying archaea and bacteria assuming one and two 16S rRNA gene copies per cell, respectively (Pei et al. 2010).

### 2.4 Parameter Estimation and Numerical Methods

The process‐based model was calibrated with Bayesian parameter‐estimation to account for uncertainty. Prior distributions were defined according to literature values of the parameters (Table S6). We estimated one set of parameters for each river segment (upstream, midstream, downstream) and depth (5 cm, 15 cm) independently, that is, six sets. To calculate the likelihood for Bayesian inference, we used measured concentrations of ammonium and nitrate as well as *amoA* and 16S rRNA gene abundances. The data were assumed to follow a normal distribution centred about simulated concentrations (ammonium) or log‐transformed concentrations or abundances (nitrate, gene counts). The log‐transformation was applied to remove the concentration‐dependence of measurement standard deviations in the nitrate and gene data. We then assumed a constant measurement error that was identical for all treatments and estimated it jointly with reaction parameters. Nitrate and *amoA* data contain information about nitrification rates and bacterial growth that is necessary to constrain the model predictions. However, ammonium data were much more abundant, leading to a model fit that would be biassed towards a better fit of ammonium concentrations. Therefore, we weighted the data standard deviations by the number of measurements per data type in each microcosm, giving larger weights to scarcer measurements.

The reaction model represents a system of ordinary differential equations (ODEs) that we solved with the Python package Sunode (Seyboldt 2021) using the backward‐differentiation‐formula (BDF) solver as implemented in the SUNDIALS library (Hindmarsh et al. 2005). The posterior distribution is sampled with the No U‐Turn Sampler (NUTS) using the Python package PyMC (version 5.10.4) (Abril‐Pla et al. 2023). We ran two independent Markov chains, generating 1000 samples in each chain. We assessed convergence by comparing within‐chain and between‐chain variance with the $\hat{R}$‐criterion (Vehtari et al. 2021). Details of the Bayesian parameter‐estimation method for the reaction model are shown in the Supporting Information.

### 2.5 Molecular Analyses and Sequence Data Handling

All procedures for phenol–chloroform‐based DNA extraction, the generation and sequencing of prokaryote 16S rRNA gene and bacterial *amoA* gene amplicons, as well as sequence data handling are described in the Supporting Information.

## 3 Results

### 3.1 In Situ Community Composition of Streambed Microbiomes

**FIGURE 2:**
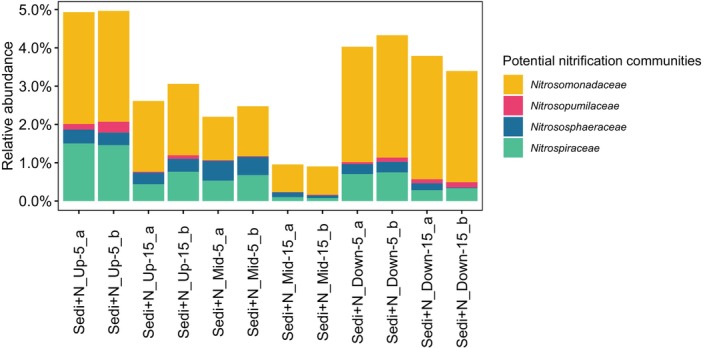
Relative abundances of taxa affiliated with well‐known nitrifiers in amplicon libraries of streambed sediments before microcosm incubation. Shown are members of the AOB (*Nitrosomonadaceae*) and AOA (*Nitrosopumilaceae, Nitrosphaeraceae*) and the nitrite‐oxidising bacteria (*Nitrospiraceae*).

Prior to the microcosm experiment, streambed microbiota of up‐, mid‐ and downstream sections of the Schönbrunnen were analysed to infer initial in situ microbial‐community composition. All fresh samples (*T*_0_) showed similar ASV‐based Shannon diversity (*H*′ = 6.3–7.4) with insignificant depth differences, except for samples taken from the midstream section. The Shannon diversity for midstream 5 cm samples (*H*′ = 7.2) was significantly greater than midstream 15 cm samples (*H*′ = 5.1) (Figure S1). The 20 most abundant family‐level lineages took up similar proportions of the total microbiota (40%–60%) at all sampling spots before incubation (Figure S2). Potential sulphur‐oxidising populations, such as members of the *Sulfurimonadaceae* (relative abundance up to 17.7%) and *Hydrogenophilaceae* (relative abundance up to 6.4%), were among the dominant taxa in the Schönbrunnen streambed. Among presumed AOB lineages, the *Nitrosomonadaceae* appeared most abundant (relative abundance up to 3.2%, Figure 2). However, not all members of *Nitrosomonadaceae* are ammonium oxidizers and no evidence of ammonia oxidation exists for the two dominant genus‐level taxa MND1 and Ellin6067 (Figure S3). The *Nitrosomonadaceae* were generally more abundant in upstream and downstream samples (Figure 2), previously identified as net gaining sections of the stream (Jimenez‐Fernandez et al. 2022). Their abundance was also generally greater in 5 cm depths than in 15 cm depths. A similar distribution pattern was also observed for potential nitrite‐oxidising bacteria (NOB) within the *Nitrospiraceae* (dominated by reads affiliated with the genus *Nitrospira*) (Figure 2). In contrast, AOA were less abundant taxa in 16S amplicon libraries. That is, members of the *Nitrososphaeraceae* had a mean relative abundance of ~0.3%, while the *Nitrosopumilaceae* had a mean relative abundance of only < 0.1% (Figure 2). However, qPCR data showed that in situ archaeal *amoA* gene copies (3.9 × 10^5^–2.1 × 10^6^ gww^−1^ sediment) exceeded bacterial *amoA* gene copies (3.5 × 10^4^–3.6 × 10^5^ gww^−1^ sediment). This discrepancy likely reflects the greater accuracy of qPCR for quantifying functional genes compared to 16S rRNA sequencing, especially given that not all taxa classified as potential AOB are necessarily capable of ammonia oxidation.

### 3.2 Microcosm Incubation Experiments

To further dissect not just the longitudinal distribution, but also the potential activity of streambed ammonia‐oxidising populations, a microcosm experiment was set up with distinct chemical inhibitors. Potential nitrification rates and population growth were analysed with the process‐based reaction model described above over a 14‐day incubation period.

#### 3.2.1 Ammonia Oxidation

**FIGURE 3:**
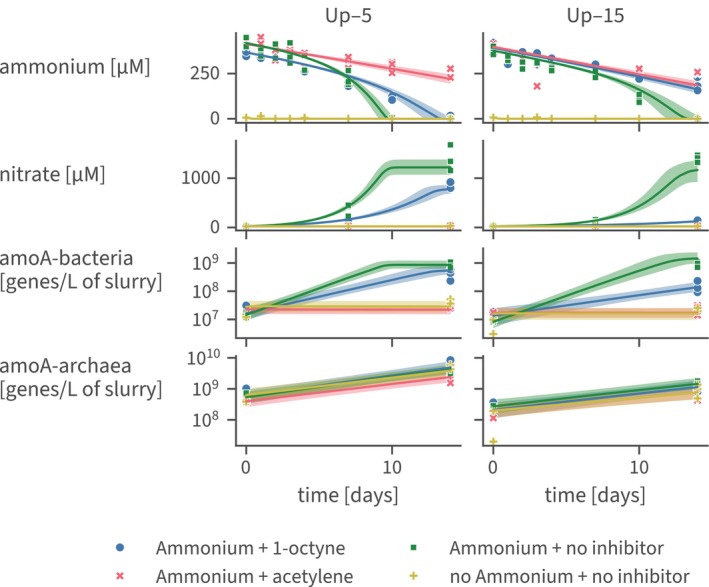
Measured (symbols) and modelled (lines) concentrations of ammonium, nitrate and bacterial/archaeal *amoA* genes in microcosms with upstream (5 and 15 cm) sediment samples. Shaded areas span between the 10th and 90th percentile and lines represent the median of the simulated concentrations. Measurements from different biological replicates are indicated by separate markers. Measured and simulated time series for midstream and downstream sediment samples were largely consistent and are shown in Figures S7 and S8.

Initial NH_4_^+^ concentrations in ammonium‐amended microcosms were around 0.4 mM, matching the upper range observed at the Schönbrunnen field site (Table S1). This value is slightly lower than the nominal added concentration of 0.5 mM, reflecting the rapid, abiotic adsorption of ammonium to the sediment's cation exchange sites immediately following the spike. In microcosms with ammonium but no inhibitors, indicating potential in situ nitrification, both ammonium depletion and nitrate production were observed consistently across stream segments (Figure 3). Ammonium was nearly depleted after 14 days in all samples, while nitrate levels reached up to 1.4 mM, exceeding initial ammonium levels threefold. Although nitrate formation was delayed in deeper sediment samples (15 cm), final concentrations were similar across depths.

Distinct ammonium patterns were observed in microcosms with different nitrification inhibitors (Figure 3). Microcosms treated with 6 μM acetylene (an inhibitor of autotrophic nitrification by both AOA and AOB) showed only a slight decrease of ammonium levels, with no detectable nitrate. This suggests inhibition of autotrophic nitrification and absence of nitrification by heterotrophic microorganisms. Conversely, microcosms with 4 μM 1‐octyne (inhibitor of AOB only) revealed a significant ammonium decrease in shallow (5 cm) microcosms, alongside nitrate production up to 0.9 mM. However, 15 cm microcosms with 1‐octyne exhibited only a minor ammonium decrease (~0.2 mM) and nitrate remained near the detection limit (15 μM). In controls containing only sediments, without ammonium or inhibitor amendments, as well as in those with stream water only, neither ammonium depletion nor nitrate production was detected throughout the incubation period (Figures 3, S7 and S8).

#### 3.2.2 Microbial Population Dynamics

Bacterial and archaeal 16S rRNA and *amoA* genes in microcosm samples were quantified via qPCR to assess the abundance of total microbial populations and ammonia‐oxidizers during the incubation (Figures 3 and S9; Table S4). Before incubation, bacterial 16S rRNA genes (average 2 × 10^8^ copies g_ww_^−1^ sediment) were 3–12 times more abundant than archaeal 16S rRNA genes. Initial archaeal *amoA* abundances exceeded the abundance of bacterial *amoA* genes by approximately one order of magnitude (Table S5). During incubation, archaeal *amoA* gene counts rose moderately in all treatments (by a factor of four on median), regardless of inhibitor presence. This increase suggests that AOA abundance remained relatively stable and appeared unaffected by active ammonia oxidation. In contrast, bacterial *amoA* gene abundances increased by up to two orders of magnitude in several microcosms with nitrate production, specifically in microcosms without inhibitor or with 1‐octyne (Figure S4). These patterns in *amoA* abundances and nitrate production suggest that the increase in bacterial *amoA* genes was strongly linked to active ammonia oxidation, whereas the moderate growth of AOA appeared largely independent of this process.

#### 3.2.3 Simulated Dynamics of Nitrogen and Ammonia Oxidising Populations

The reaction model served to test our conceptual understanding of processes occurring in the microcosms and to quantify the contributions of AOA and AOB to nitrification rates. Overall, the simulations reflected the patterns of measured concentrations of solutes and functional genes well (Figure 3). Model‐predicted ammonium concentrations decreased consistently over time in treatments with ammonium and no inhibitors or with 1‐octyne. The strongly reduced ammonium depletion in treatments with acetylene was also captured by the model. According to the model, the observed slight ammonium depletion was attributed to the incorporation of ammonium into microbial biomass. The model further correctly captured the observed increase of nitrate in the ammonium‐amended microcosms without an inhibitor or with 1‐octyne, including the nitrate rise to levels exceeding the initial ammonium concentration. Only in microcosms *Mid‐5* and *Down‐15*, the model underestimated final nitrate concentrations (Figures S7 and S8). This disagreement might be caused by our relatively simple description of the release of surplus nitrogen from the sediment (as further discussed below). However, it remains unclear why the model was unable to represent nitrate data of microcosms *Mid‐5* and *Down‐15* just as well as for the other microcosms, despite remarkably similar observed patterns. Simulated and measured growth dynamics of AOA and AOB also matched well. The model particularly reproduced the strong growth of AOB in the treatments with high nitrate production and the moderate growth of AOA in all treatments (regardless of active ammonia oxidation).

#### 3.2.4 Kinetic Parameters of Ammonium Oxidation

A comparison of the prior and posterior parameter distribution revealed how well the measurements constrained the model parameters controlling ammonia oxidation. The posterior distributions of half‐saturation constants for archaeal ammonia oxidation, the 1‐octyne‐inhibition parameter for AOA and the microbial decay constant were nearly identical to prior distributions (Figure S6 and Table S6), indicating that only little information was gained from the data. Other posterior distributions, however, were characterised by much narrower parameter ranges and partially strong shifts of the distributions compared to prior parameter distributions. For example, maximum ammonia oxidation rates shifted to larger values for AOB, but to smaller values for AOA. Also, *K*_AOB_ shifted to smaller values, which were in the lower range of reported experimental values (Jung et al. 2021). Additionally, the distribution of $f_{\text{inhib}}$ for AOB shifted towards larger values, corresponding to less inhibition than what we originally expected.

No substantial differences in parameter estimates were observed between microcosms from different stream segments (Figure S6). Parameter differences based on sediment depth were small, but noticeable for some parameters. For example, 1‐octyne inhibition (measured by estimated $f_{\text{inhib}}$) was stronger in 15 cm‐microcosms compared to 5 cm‐microcosms. Furthermore, the maximum specific ammonia oxidation rate of AOB was slightly smaller in 15 cm‐microcosms, reflecting the observed slower ammonium consumption. Another factor supporting the faster oxidation of ammonium in 5 cm microcosms was the generally higher initial abundances of AOB.

#### 3.2.5 Contributions of AOA and AOB to Ammonia Oxidation

**FIGURE 4:**
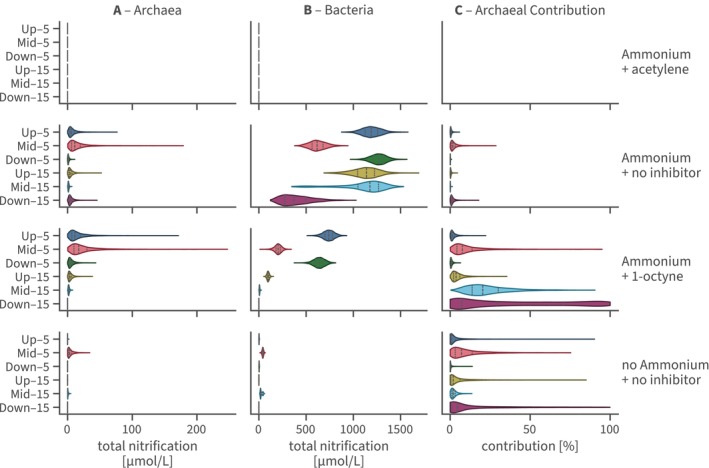
Posterior model estimates of nitrification rates in the different microcosms for archaea (A) and bacteria (B), integrated over the duration of the experiment. (C) Contribution of archaea to total nitrification. Note that the scaling of the *x*‐axis differs between (A) and (B).

The model‐based posterior estimates of total nitrification, that is, nitrification rates integrated over time (Figure 4), revealed the contributions of AOA and AOB to nitrification in the microcosms. AOB appeared almost exclusively responsible for ammonia oxidation, whereas AOA played a very minor role, particularly, when ammonium was added to the microcosms. The calculated median contribution of AOB in the treatments amended with ammonium and without inhibitors exceeded 97% for all sediment samples. When AOB were partly inhibited by 1‐octyne, the model‐estimated contribution of AOA to nitrification was no longer negligible, but still much lower than that of AOB (median values for AOA range from 1% to 39%). Archaeal nitrification also reached a higher contribution in microcosms where no ammonium was added (median values between 0.4% and 15%). It should be noted that the estimated contribution of archaea to nitrification in the treatments amended with ammonium was particularly high in those microcosms, where the model could not fit nitrate production very well (e.g., *Mid‐5* and *Down‐15*). In these microcosms, nitrification estimates should thus be treated with caution, as the mismatch of nitrate data indicates that some processes influencing nitrate dynamics were not well described by the model.

### 3.3 Diversity and Composition of AOB Gene Pools in Microcosms

#### 3.3.1 Richness and Diversity Indices

**FIGURE 5:**
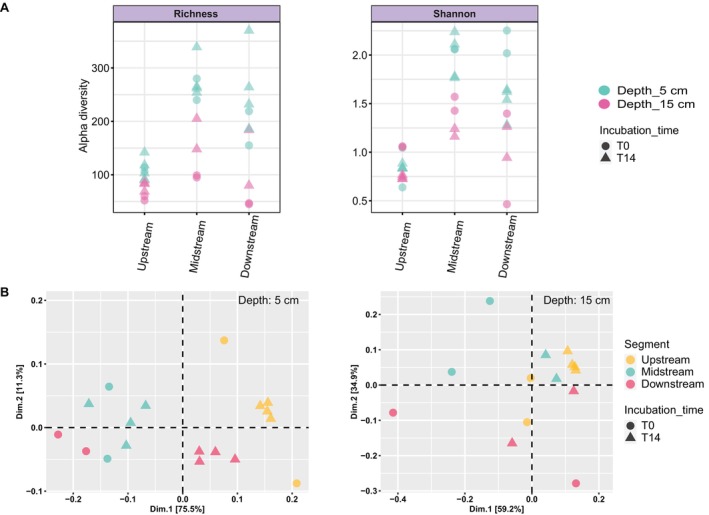
(A) Observed richness and Shannon diversity indices of bacterial *amoA* OTUs in 5 and 15 cm sediments before (*T*_0_) and after (*T*_14_) incubation, including all treatments. (B) PCA plots of bacterial *amoA* OTUs in 5 and 15 cm sediments of different stream segments (upstream, midstream and downstream) before (*T*_0_) and after incubation (*T*_14_).

Given the dominant role of AOB in ammonia oxidation in our sediments, we analysed the diversity of AOB gene pools by sequencing *amoA* amplicons. In total, 764 OTUs were generated at a 95% similarity cutoff. Richness and Shannon indices were assessed for each stream segment before (*T*_0_) and after (*T*_14_) incubation (Figure 5). In general, *amoA* diversity and richness of AOB were higher at 5 cm depth than at 15 cm. Upstream samples had the lowest richness (mean = 93 OTUs) compared to midstream (mean = 219) and downstream samples (mean = 178). Richness was equal or higher after incubation, with downstream samples from 5 cm depth amended with ammonium showing the highest richness (370 OTUs).

Shannon diversity showed similar trends (Figure 5), being highest in midstream and downstream 5 cm samples. We observed a decrease in diversity over the incubation for upstream 15 cm, midstream 15 cm and downstream 5 cm samples, suggesting a filtering of nitrifier populations in our experimental setup. The Shannon diversity values observed by us are on the lower end of values reported for other river or estuarine sediments (Zhang et al. 2020, 2014; Damashek et al. 2014; Cao et al. 2011). There were no treatment‐specific patterns in richness or diversity indices.

#### 3.3.2 Shifts in Community Composition

The composition of *amoA* gene pools varied across stream segments, depths and incubation time (Figure 5B). Initially, 5 cm upstream communities clustered separately from midstream and downstream samples. After incubation, upstream, midstream and downstream samples formed three distinct clusters, with community shifts most marked in upstream and downstream samples, suggesting ammonium amendment triggered changes in AOB communities for these segments but not for midstream samples. Since upstream and downstream sites represent stream segments with a discharge of sulphur‐rich groundwater to the stream (Wang et al. 2022; Jimenez‐Fernandez et al. 2022) and sulphide concentrations in the μM range are known to significantly inhibit nitrification (Joye and Hollibaugh 1995), water chemistry could impact AOB community diversity and composition in the Schönbrunnen stream sediments. At 15 cm depth, *amoA* gene pools were similar across samples at *T*_14_, despite initial heterogeneity. Overall, no consistent community shifts were observed between treatment groups (data not shown).

#### 3.3.3 Dominant OTUs and Phylogenetic Affiliations

**FIGURE 6:**
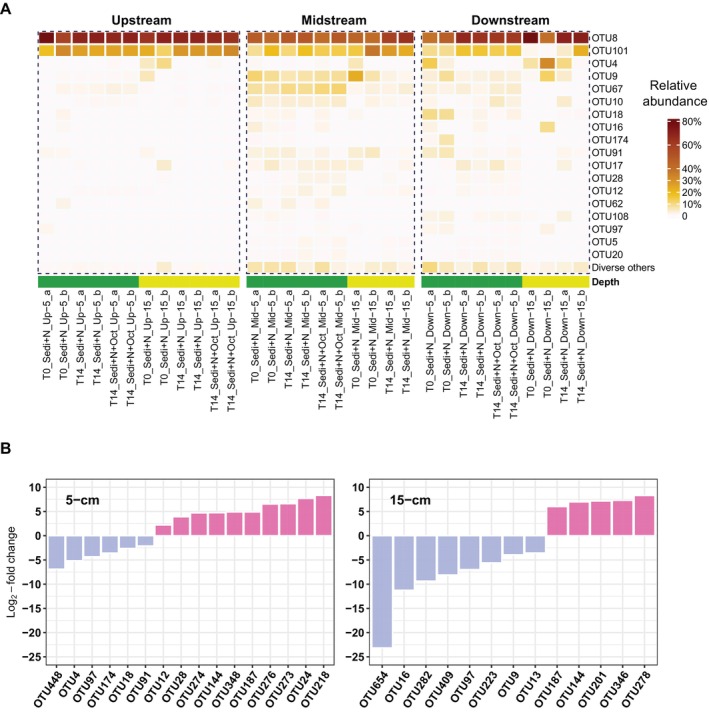
(A) Relative abundance of bacterial *amoA* OTUs before (*T*_0_) and after (*T*_14_) incubation in different treatments, stream segments and depths of replicate microcosms. Two treatments were included: sediment amended with ammonium (Sedi + N) and sediment amended with ammonium and 1‐octyne (Sedi + N + OCT). Sample names on *x*‐axes indicate original sampling locations. ‘a’ and ‘b’ indicate duplicate microcosms. OTUs of low abundance were merged into ‘diverse others’ (cumulative abundance in all samples < 2%). (B) Log_2_‐fold change of bacterial *amoA* OTUs significantly differing in abundance before (*T*_0_) and after (*T*_14_) microcosm incubation (*p*_adj_ < 0.05, Wald test with Benjamini–Hochberg correction) in 5 and 15 cm sediments, including treatments ‘Sedi’ + N and ‘Sedi + N + OCT’.

**FIGURE 7:**
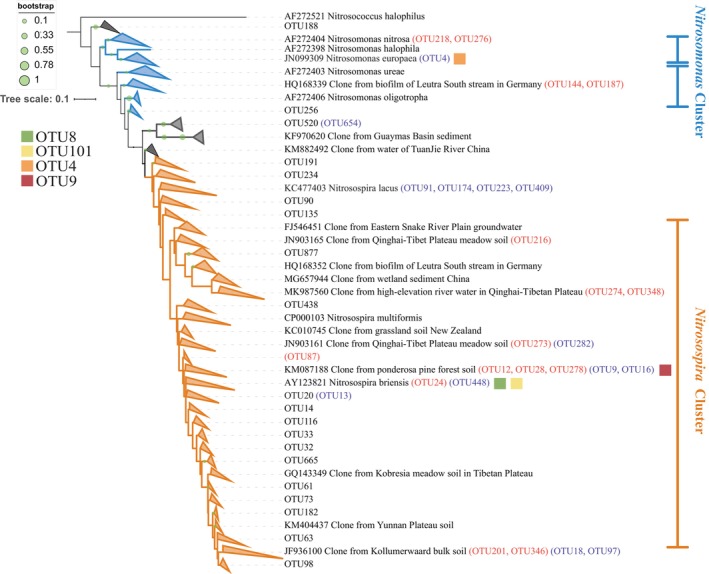
Maximum likelihood tree generated from a nucleotide‐based alignment of all bacterial *amoA* OTUs detected in this study. Coloured squares indicated phylogenetic affiliations to the four most abundant OTUs from Figure 6A. Blue colour clusters are at least 85% identical to *Nitrosomonas* isolates, whereas orange colour clusters are at least 85% identical to *Nitrosospira* isolates. OTU names written in red colour represent OTUs whose abundance positively changed after incubation as shown in Figure 6B, whereas OTU names in purple colour decreased in abundance. Within each triangle cluster of the tree, several OTUs were collapsed if average similarities were > 85%.

Among the 764 *amoA* OTUs, four (OTU8, OTU101, OTU4 and OTU9) accounted for the largest share (87.6% mean cumulative relative abundance across samples), with OTU8 (mean relative abundance of 60%) and OTU101 (19.4%) being the most prevalent in almost all samples. OTU4 was more abundant in *T*_0_ samples, while OTU9 was more abundant in midstream and downstream samples (Figure 6). Phylogenetic analysis revealed that 57 out of 764 OTUs clustered with *Nitrosomonas*, while the rest were grouped with *Nitrosospira*. Notably, OTU8, OTU101 and OTU9 were *Nitrosospira* or *Nitrosospira*‐like sequences (Figure 7) related to *N. briensis* (87%–89% identity), whereas OTU4 was identified as *Nitrosomonas* sequences (100% identical to *N. europaea* ).

The abundance of 13 *amoA* OTUs increased after incubation in ammonium‐amended microcosms (Figure 6B). An increase of OTU abundance was observed more frequently in 5 cm samples than in 15 cm samples, likely reflecting the faster ammonia oxidation rates observed at 5 cm depth. Two OTUs affiliated with *Nitrosomonas* (OTU144 and OTU187) were more abundant post‐incubation at both depths, while most depth‐specific changes occurred for OTUs clustered with *Nitrosospira* (Figures 6 and 7). Interestingly, OTU9 and OTU4 decreased in abundance post‐incubation, suggesting limited adaptation to microcosm conditions despite their likely relevance in situ.

## 4 Discussion

Taken together, the process measurements, modelling results and molecular data provide complementary insights into ammonia‐oxidizer abundance and activity and are discussed below in relation to the questions that motivated this study. The relative contributions of AOA and AOB are evaluated based on the model‐derived quantification of nitrification rates (Section 3.2.5) and further interpreted in Section 4.4. The identity and response of ammonia‐oxidising taxa to ammonium pulses are inferred from the combined analysis of *amoA* gene abundances and sequencing data (Sections 3.1 and 3.3) and discussed in Section 4.5. Finally, we synthesise these results to assess whether *amoA* gene abundances reflect population‐specific nitrification activity in Section 4.4 and in the Conclusions.

### 4.1 Growth of AOA Independent of Ammonium Oxidation

The growth of AOA independent of ammonium oxidation was unexpected, since AOA are considered specialists whose energy metabolism relies on ammonia oxidation. However, this behaviour has also been observed in an experimental study with an AOA enriched from an arctic soil (Alves et al. 2019) and there is evidence that *amoA*‐carrying Thaumarchaeota from a wastewater treatment plant used reactions for energy conservation other than chemolithoautotrophic ammonia oxidation (Mußmann et al. 2011). A growing body of genomic studies further suggests that AOA are metabolically versatile (Wright and Lehtovirta‐Morley 2023). Potential alternative metabolisms predicted from AOA genomes include nitrate reduction (Zheng et al. 2024), fermentation of amino acids (Daebeler et al. 2018) and hydrogen oxidation (Daebeler et al. 2018; Abby et al. 2018), but experimental confirmation is pending. Notably, Zheng et al. (2024) present genomic evidence indicating that Ca. *Nitrosomirales* AOA may be able to utilise formate as an alternative electron donor. Including the growth term of AOA independent of ammonium oxidation in the model was supported by the observation of AOA growth in acetylene‐inhibited and no‐ammonium treatments. However, the design of our study does not allow us to verify any specific pathway of a potential alternative metabolism. Targeted enrichment or isotope‐labelling experiments would be needed to test whether these alternative metabolic pathways proposed from genomic studies are active in the investigated sediments.

To test whether the observed AOA growth could still be explained by very low ammonia oxidation rates, we estimated the minimum empirical growth yield that would be required to explain the increase of archaeal *amoA* genes for the ‘no ammonium + no inhibitor treatment’ while nitrate stays below the detection limit of 15 μM (since nitrate was not detected). Our calculation indicates that the minimum required empirical growth yield (8.4 × 10^13^ cells/mol of ammonia) falls within the range of reported growth yields for AOA (Jung et al. 2011). Thus, growth of AOA from extremely low ammonia‐oxidation rates cannot be ruled out. However, the latter cannot explain the increase in AOA abundance in the acetylene treatment, in which ammonia oxidation is completely inhibited.

To further test if the model could reproduce the data also when disregarding an alternative growth process, we set up a second model variant. In this model, AOA can only grow when oxidising ammonia, but we allowed for partial acetylene inhibition that could explain growth of AOA in the acetylene treatment. The simulation results with this alternative model were similar compared to the original model (results not shown). However, the alternative model could not reproduce the increase of archaeal *amoA* counts observed in the microcosms without ammonium addition and it also could not fully explain the respective increase in the acetylene treatment (in contrast to the original model). Our data and modelling results thus suggest an alternative growth process for AOA, but additional enrichment or labelling experiments would be necessary to confirm this.

### 4.2 Release of Ammonium From the Sediment

We observed that the formation of nitrate, which is the major product of nitrification, over the incubation largely exceeded the initial concentration of ammonium amended to the microcosms of all stream segments (Figures 3, S7 and S8). Nitrification is expected to convert ammonium to equal molar concentrations of nitrate (Kuypers et al. 2018). Therefore, an additional source of nitrogen, possibly organic nitrogen in the sediment, must have been used as a substrate for nitrification, explaining the overproduction of nitrate. Hence, overall ammonia oxidation rates can be underestimated when relying on the depletion of added ammonium alone for calculations. Unlike ammonium, which is also produced by alternative pathways such as urea hydrolysis or the mineralisation of other organic nitrogen, the oxidative formation of nitrate is strictly attributed to nitrification under oxic conditions (Sonthiphand and Neufeld 2014; Levičnik‐Höfferle et al. 2012). Therefore, nitrate production rates were selected as the key indicator of nitrification activity in our experiment and modelling approach.

The model represented the release of additional nitrogen by an ammonium‐production term that depended linearly on the nitrification rate (see Equation 4). This description was based on the observation that additional nitrate was produced only in microcosms with active nitrification. In most microcosms, this simple empirical relationship seemed to be sufficient to describe the release process since the disproportionate increase of nitrate observed in the data was reproduced by our model. The median model‐estimated ammonium release ranged between 0.38 (*Mid‐5*) and 0.85 (*Up‐15*) mol of ammonium released per mol of ammonium nitrified. The fact that the model underestimated the pronounced increase of nitrate in microcosms *Mid‐5* and *Down‐15* suggests that the model does not describe the ammonium release appropriately and a more mechanistic process description would be required in these cases.

The model itself does not account for any details on the source of the additional nitrogen or the mechanisms of its release. However, members within *Nitrosomonas*, *Nitrosospira* and *Nitrososphaera*, among others, are known to degrade organic nitrogen compounds, hydrolyze urea with ureases and thus produce additional ammonia for nitrification (Burton and Prosser 2001; Tourna et al. 2011). Moreover, not only ammonia oxidizers, but also NOB can produce ammonia from urea and benefit by receiving additional nitrite from AOB or AOA. This process was described as reciprocal feeding (Koch et al. 2015). Considering that nitrate concentrations did not increase when microcosms were not amended with ammonium, the described interaction between NOB and ammonia‐oxidizers is a likely explanation for the surplus ammonium released in our study. However, further analyses of total nitrogen and total organic‐nitrogen pools would have been necessary to comprehensively delineate these mechanisms and provide the constraints needed for a model that represents them explicitly.

### 4.3 Incomplete Inhibition of AOB by 1‐Octyne

The increase of bacterial *amoA* genes in treatments with 1‐octyne, concomitant with an increase in nitrate concentrations, suggests that 1‐octyne in microcosms only partially inhibited ammonia oxidation by AOB. To explore this hypothesis, we incorporated a parameter for the degree of inhibition by 1‐octyne ($f_{\text{inhib}}$) into the reaction model and inferred its value from the data. The estimated values of $f_{\text{inhib}}$ were larger than zero, indicating incomplete inhibition of ammonia oxidation by AOB (Figure S6). The median values of inhibited rates ranged between 12% (*Mid‐15*) to 62% (*Up‐*5) of the uninhibited rate. Overall, the inhibition was stronger in the sediments from 15 cm depth than from 5 cm. This observation contradicted previous reports suggesting that 1‐octyne is capable of robustly inhibiting AOB, both in pure culture cell suspensions and in soil microcosms (Taylor et al. 2013; Hink et al. 2017). This discrepancy likely reflects conditions specific to our experimental system, rather than a general limitation of 1‐octyne as an inhibitor. The incomplete inhibition of AOB might be attributed to the complexity and specific composition of ammonia‐oxidising communities in the investigated sediments or to limited bacterial exposure to 1‐octyne due to sorption of the inhibitor with this specific sediment type.

This points to the importance of experimental design when 1‐octyne is applied to determine contributions of AOA and AOB to nitrification. Here, the growth of AOB in the presence of 1‐octyne helped us to infer incomplete inhibition, but this is only feasible in studies where mixotrophic AOB do not play a vital role. In future work, ^15^N‐labelled substrates could help to further determine the degree of inhibition of AOB by 1‐octyne in environmental samples and to better identify the contributions of either AOA or AOB to ammonia oxidation when AOA or AOB may also grow through other processes than ammonia oxidation. In addition, future work should include experiments with gradients of the inhibitor concentration to ensure efficacy of 1‐octyne for the investigated microbial community and matrix.

In this study, the combination of partial 1‐octyne‐inhibition of AOB, the growth of AOA independent of nitrate production and the apparent presence of additional nitrogen sources very much complicated the direct inference of nitrification rates, population‐specific growth rates and the contributions of AOA and AOB to gross nitrification from experimental data alone. In our study, linking the experimental data with a process‐based reaction model was a clear asset to disentangle and quantify the impact of these factors and to better estimate population‐specific nitrification rates and uncertainties.

### 4.4 Contributions of AOA, AOB, and Comammox to Ammonia Oxidation

Our results show that ammonia oxidation in the investigated stream sediments is driven primarily by AOB under the experimental conditions. In the following, we discuss how this result relates to environmental factors and AOB physiology and how potential comammox activity may influence its interpretation.

Ammonium concentrations in the low μM range are considered as growth‐limiting for AOB (Bollmann et al. 2002). Previous studies on terrestrial samples and pure cultures indicated that AOA are better adapted to low ammonium availability, while AOB outcompete AOA at higher ammonium concentrations (Jia and Conrad 2009; Martens‐Habbena et al. 2009). Our microcosms were amended with an ammonium concentration well within the range found in situ (up to 5.5 mM were measured in the field, Table S1) and such concentrations also reflect episodic high‐load conditions in agricultural streams, where runoff pulses can transiently deliver elevated ammonium inputs. However, several groundwater samples also showed lower ammonium concentrations. To test the contributions of archaeal and bacterial nitrifiers to nitrification at more limiting concentrations, we calculated ammonium oxidation rates based on the posterior samples of the kinetic rate parameters from the microcosm experiments, but using lower ammonium concentrations (10 μM) and the *amoA* abundances of the in situ samples. Under these conditions, ammonium oxidation by bacteria is still at least one order of magnitude larger than by archaea (Figure S5). These results suggest that AOB dominate nitrification in the investigated agricultural stream system. In contrast, AOA were not found to be major players in ammonia‐oxidation in Schönbrunnen sediments, even though they were of predominant abundance in situ (as per *amoA* gene qPCR). Besides concentration effects, another possible explanation of this discrepancy could be that AOA did not actually express *amoA* genes under the conditions of our experiment. The moderate population growth of AOA, independent of ammonia oxidation, could indicate that the AOA in the streambed use an alternative growth metabolism.

Our study does not identify the specific role of comammox bacteria in nitrification due to several methodological constraints. The primers used in qPCR and amplicon sequencing target *amoA* gene sequences of AOB, preventing the detection of comammox bacteria. Consequently, our qPCR measurements of AOB and AOA did not account for comammox. Without direct measurements of comammox abundance, we also lacked data to constrain their growth parameters and have therefore excluded them from our model.

In terms of chemical inhibition, existing literature supports the complete inhibition of ammonia oxidation in comammox by acetylene, but the effect of 1‐octyne is less certain. Some studies indicate that 1‐octyne does not affect comammox *Nitrospira*, while others suggest an inhibitory effect (Li et al. 2019; Lin et al. 2023). If comammox were active in our microcosms and unaffected by 1‐octyne, the observed increase in nitrate concentrations in the 1‐octyne‐treated microcosms might be explained by comammox activity rather than by partially inhibited AOB. However, this would not explain the increase in AOB‐*amoA* gene abundance in these treatments.

If comammox contributed to ammonia oxidation in our microcosms—an aspect not included in our model—our estimated maximum cell‐specific oxidation rates would be too high, while growth yields would be too low. Overall, the role of comammox in the Schönbrunnen sediments remains uncertain. Comammox could be responsible for a portion of the ammonium oxidation that our model currently attributes to AOB. Thus, it might be more accurate to refer to the combined contribution of both AOB and comammox in nitrification across our results. To resolve the uncertainty caused by the lack of comammox detection, future studies should employ comammox‐specific primer sets alongside AOA‐ and AOB‐targeted assays to better resolve the role of comammox in streambed nitrification.

### 4.5 Ecological Context

Our findings indicating that *Nitrosomonas* and *Nitrosospira* spp. were the primary AOB in the Schönbrunnen stream are consistent with observations in other freshwater systems (Bernhard and Bollmann 2010). While *Nitrosomonas europaea* (e.g., OTU4) is considered less competitive under ammonium‐limited conditions (Bollmann et al. 2002; Koops and Pommerening‐Röser 2001), *Nitrosospira* was predominant in low‐ammonium marine sediments (Lagostina et al. 2015), suggesting high ammonium affinity. *K*_s_‐values for AOB estimated with the reaction model ranged between 5 and 60 μM (10th and 90th percentile aggregating over all posterior samples, stream segments and depths; see also Figure S6), consistent with the lower end of values reported for *Nitrosospira* spp. and *Nitrosomonas* strains (between 10 and 100 μM) (Jung et al. 2021).

Our high‐throughput sequencing enabled the detection of a much greater number of *amoA* OTUs (observed richness) compared to other studies of riverine systems (Damashek et al. 2014). However, the MiSeq paired‐end 2 × 250 bp sequencing approach was not able to cover the full length of bacterial *amoA* gene amplicons, so that the presented phylogenetic analysis cannot capture the complete diversity of AOB (Aigle et al. 2019). Therefore, phylogenetic assignments at the genus level should be interpreted with caution. Nonetheless, our study provides valuable insights into the diversity and distribution of AOB *amoA* genes in a lower‐order agricultural stream, relevant to understanding nitrogen cycling under anthropogenic influences.

## 5 Conclusions and Environmental Significance

We used an integrated approach combining field observations, microcosm experiments and process‐based modelling to investigate the contributions of discrete microbes to ecosystem processes. Despite a much higher in situ abundance of AOA, our study clearly attributed a higher reactivity towards incoming ammonium pulses to AOB and potentially comammox. Particularly *Nitrosomonas* and *Nitrosospira* populations were identified as important contributors to nitrification in streambed sediments. Even though we observed pronounced differences in AOB community diversity and abundance in situ, overall ammonia‐oxidation rates and associated microbial kinetics were comparable for microcosms of distinct stream segments. This suggests that environmental filtering by a pulse of ammonium and sufficient oxygen supply readily selected for similar populations of AOB, irrespective of their original abundance in situ. Future studies should address why the observed release of additional nitrogen from the sediments only occurred when nitrification was active and what mechanisms could lead to a growth of AOA independent of ammonium oxidation. Our findings also show that using functional‐gene abundance alone is not sufficient to infer the functional relevance of ammonia oxidizers in situ. Considering that lower‐order streams are well connected not only with the surrounding soils and groundwater, but also with downstream riverine ecosystems, our results substantiate the role of AOB and potentially comammox bacteria, but not archaea, in controlling the oxidative efflux of nitrate originally stemming from agricultural ammonium inputs. We recommend considering these small streams and the microbial ecology of their streambed when improving current management concepts for the fluxes of nitrogen in landscapes dominated by agricultural land use.

## Author Contributions

**Olaf A. Cirpka:** funding acquisition, supervision, writing – review and editing. **Aileen Jung:** conceptualization, data curation, investigation, writing – original draft. **Daniel Straub:** data curation, writing – review and editing. **Zhe Wang:** conceptualization, investigation, data curation, visualization, writing – original draft, writing – review and editing. **Anna Störiko:** conceptualization, data curation, formal analysis, software, visualization, writing – original draft, writing – review and editing. **Holger Pagel:** supervision, writing – review and editing. **Tillmann Lueders:** conceptualization, funding acquisition, supervision, writing – review and editing.

## Funding

This work was funded by the German Research Foundation (DFG) (CRC 1253, RTG 1829 and STR 481/12‐1).

## Conflicts of Interest

The authors declare no conflicts of interest.

## Supporting information


**Table S1:** Schönbrunnen stream discharge (*Q*), groundwater table depth *d* (in metres below ground level) and major water chemistry parameters in both the stream water and adjacent groundwater measured in the sampling month (June 2020).
**Table S2:** Simulated stream water medium modified from a previous study (de la Torre 2008).
**Table S3:** Primers and annealing temperatures (*T*) for qPCR amplification of bacterial and archaeal 16S rRNA genes and *amoA* genes.
**Table S4:** Bacterial and archaeal 16S rRNA gene copy numbers per gramme wet sediment before (Day 0) and after (Day 14) the microcosm incubation as determined by quantitative PCR.
**Table S5:** Bacterial and archaeal *amoA* gene copy numbers per gramme wet sediment before (Day 0) and after (Day 14) the microcosm incubation as determined by quantitative PCR.
**Figure S1:** The in situ Shannon diversity index (*H′*) in all sediment samples.
**Figure S2:** Relative abundance of Top 20 most abundant microbial lineages at the family level detected in Schönbrunnen sediments prior to the microcosm incubation (*T*_0_).
**Figure S3:** Relative abundance of *Nitrosomonadaceae*‐affiliated ASVs clustered at the genus level before and after the microcosm incubation.
**Figure S4:** Increase of *amoA* gene concentrations from Days 0 to 14.
**Figure S5:** Ammonium oxidation rates calculated with an ammonium concentration of 10 μM, using the *amoA* concentrations measured in the in situ sediment samples the posterior samples of kinetic parameters.
**Figure S6:** Posterior distributions of model parameters describing the oxidation of ammonium and growth of AOA and AOB for microcosms from different stream segments and depths.
**Table S6:** Model parameters of the reaction model and the corresponding prior distributions for the Bayesian estimation of parameter values.
**Table S7:** Parameters for the reaction model of the microcosm experiments that were set to fixed values.
**Figure S7:** Measured and simulated time series of ammonium, nitrate and bacterial and archaeal *amoA* gene concentrations for microcosm from midstream 5 cm and 15 cm sediment samples.
**Figure S8:** Measured and simulated time series of ammonium, nitrate and bacterial and archaeal *amoA* gene concentrations for microcosm from downstream 5 and 15 cm sediment samples.
**Figure S9:** Simulated and measured 16S rRNA gene counts (left: archaea, right: bacteria) for all stream segments and depths.

**Table S8:** Convergence statistics and summary statistics of the parameter posterior distribution.

## Data Availability

The source code of the reaction model and the experimental data used for parameter estimation are publicly available on Zenodo (https://doi.org/10.5281/zenodo.17087314) (Störiko et al. 2025). All original sequence data have been deposited in the European Nucleotide Archive (ENA) under study accession number PRJEB103028 and in the NCBI BioProject database under accession number PRJNA1492701.

## References

[emi70369-bib-0001] Abby, S. S. , M. Melcher , M. Kerou , et al. 2018. “ *Candidatus* Nitrosocaldus Cavascurensis, an Ammonia Oxidizing, Extremely Thermophilic Archaeon With a Highly Mobile Genome.” Frontiers in Microbiology 9: 28. 10.3389/fmicb.2018.00028. 29434576 PMC5797428

[emi70369-bib-0002] Abril‐Pla, O. , V. Andreani , C. Carroll , et al. 2023. “PyMC: A Modern, and Comprehensive Probabilistic Programming Framework in Python.” PeerJ Computer Science 9, no. September: e1516. 10.7717/peerj-cs.1516. PMC10495961 37705656

[emi70369-bib-0003] Aigle, A. , J. I. Prosser , and C. Gubry‐Rangin . 2019. “The Application of High‐Throughput Sequencing Technology to Analysis of amoA Phylogeny and Environmental Niche Specialisation of Terrestrial Bacterial Ammonia‐Oxidisers.” Environmental Microbiomes 14, no. 1: 3. 10.1186/s40793-019-0342-6. PMC7989807 33902715

[emi70369-bib-0004] Alexander, R. B. , E. W. Boyer , R. A. Smith , G. E. Schwarz , and R. B. Moore . 2007. “The Role of Headwater Streams in Downstream Water Quality.” Journal of the American Water Resources Association 43, no. 1: 41–59. 10.1111/j.1752-1688.2007.00005.x. 22457565 PMC3307624

[emi70369-bib-0005] Alves, R. J. E. , M. Kerou , A. Zappe , et al. 2019. “Ammonia Oxidation by the Arctic Terrestrial Thaumarchaeote *Candidatus* Nitrosocosmicus Arcticus Is Stimulated by Increasing Temperatures.” Frontiers in Microbiology 10: 1571. 10.3389/fmicb.2019.01571. 31379764 PMC6657660

[emi70369-bib-0006] Arango, C. P. , and J. L. Tank . 2008. “Land Use Influences the Spatiotemporal Controls on Nitrification and Denitrification in Headwater Streams.” Journal of the North American Benthological Society 27, no. 1: 90–107. 10.1899/07-024.1.

[emi70369-bib-0007] Bernhard, A. E. , and A. Bollmann . 2010. “Estuarine Nitrifiers: New Players, Patterns and Processes.” Estuarine, Coastal and Shelf Science 88, no. 1: 1–11. 10.1016/j.ecss.2010.01.023.

[emi70369-bib-0008] Blann, K. L. , J. L. Anderson , G. R. Sands , and B. Vondracek . 2009. “Effects of Agricultural Drainage on Aquatic Ecosystems: A Review.” Critical Reviews in Environmental Science and Technology 39, no. 11: 909–1001. 10.1080/10643380801977966.

[emi70369-bib-0009] Bollmann, A. , M. J. Bär‐Gilissen , and H. J. Laanbroek . 2002. “Growth at Low Ammonium Concentrations and Starvation Response as Potential Factors Involved in Niche Differentiation Among Ammonia‐Oxidizing Bacteria.” Applied and Environmental Microbiology 68, no. 10: 4751–4757. 10.1128/AEM.68.10.4751-4757.2002. 12324316 PMC126422

[emi70369-bib-0010] Bouwman, A. F. , G. Van Drecht , J. M. Knoop , A. H. W. Beusen , and C. R. Meinardi . 2005. “Exploring Changes in River Nitrogen Export to the World's Oceans.” Global Biogeochemical Cycles 19, no. 1: GB1002. 10.1029/2004GB002314.

[emi70369-bib-0011] Burton, S. A. Q. , and J. I. Prosser . 2001. “Autotrophic Ammonia Oxidation at Low pH Through Urea Hydrolysis.” Applied and Environmental Microbiology 67, no. 7: 2952–2957. 10.1128/AEM.67.7.2952-2957.2001. 11425707 PMC92966

[emi70369-bib-0012] Butturini, A. , T. J. Battin , and F. Sabater . 2000. “Nitrification in Stream Sediment Biofilms: The Role of Ammonium Concentration and DOC Quality.” Water Research 34, no. 2: 629–639. 10.1016/S0043-1354(99)00171-2.

[emi70369-bib-0013] Cao, H. , Y. Hong , M. Li , and J. D. Gu . 2011. “Diversity and Abundance of Ammonia‐Oxidizing Prokaryotes in Sediments From the Coastal Pearl River Estuary to the South China Sea.” Antonie van Leeuwenhoek International Journal of General and Molecular Microbiology 100, no. 4: 545–556. 10.1007/s10482-011-9610-1. PMC3190089 21717206

[emi70369-bib-0014] Cardarelli, E. L. , J. R. Bargar , and C. A. Francis . 2020. “Diverse Thaumarchaeota Dominate Subsurface Ammonia‐Oxidizing Communities in Semi‐Arid Floodplains in the Western United States.” Microbial Ecology 80, no. 4: 778–792. 10.1007/s00248-020-01534-5. 32535638

[emi70369-bib-0015] Daebeler, A. , C. W. Herbold , J. Vierheilig , et al. 2018. “Cultivation and Genomic Analysis of ‘Candidatus Nitrosocaldus Islandicus,’ an Obligately Thermophilic, Ammonia‐Oxidizing Thaumarchaeon From a Hot Spring Biofilm in Graendalur Valley, Iceland.” Frontiers in Microbiology 9: 193. 10.3389/fmicb.2018.00193. 29491853 PMC5817080

[emi70369-bib-0016] Damashek, J. , J. M. Smith , A. C. Mosier , and C. A. Francis . 2014. “Benthic Ammonia Oxidizers Differ in Community Structure and Biogeochemical Potential Across a Riverine Delta.” Frontiers in Microbiology 5: 1–18. 10.3389/fmicb.2014.00743. 25620958 PMC4287051

[emi70369-bib-0017] David, M. B. , and L. E. Gentry . 2000. “Anthropogenic Inputs of Nitrogen and Phosphorus and Riverine Export for Illinois, USA.” Journal of Environmental Quality 29, no. 2: 494–508. 10.2134/jeq2000.00472425002900020018x.

[emi70369-bib-0018] de la Torre, J. R. , C. B. Walker , A. E. Ingalls , M. Könneke , and D. A. Stahl . 2008. “Cultivation of a Thermophilic Ammonia Oxidizing Archaeon Synthesizing Crenarchaeol.” Environmental Microbiology 10, no. 3: 810–818. 10.1111/j.1462-2920.2007.01506.x. 18205821

[emi70369-bib-0019] Duff, J. H. , F. Murphy , C. C. Fuller , F. Triska , J. W. Harvey , and A. P. Jackman . 1998. “A Mini Drivepoint Sampler for Measuring Pore Water Solute Concentrations in the Hyporheic Zone of Sand‐Bottom Streams.” Limnology and Oceanography 43, no. 6: 6. http://pubs.er.usgs.gov/publication/70021295.

[emi70369-bib-0020] Duff, J. H. , and F. J. Triska . 2000. “Nitrogen Biogeochemistry and Surface–Subsurface Exchange in Streams.” In Streams and Ground Waters, edited by J. B. Jones and P. J. Mulholland , 197–220. Elsevier. 10.1016/B978-012389845-6/50009-0.

[emi70369-bib-0021] Gadkari, D. 1984. “Influence of the Herbicides Goltix and Sencor on Nitrification.” Zentralblatt für Mikrobiologie 139, no. 8: 623–631. 10.1016/s0232-4393(84)80056-6. 4090765

[emi70369-bib-0022] Galloway, J. N. , A. R. Townsend , J. W. Erisman , et al. 2008. “Transformation of the Nitrogen Cycle: Recent Trends, Questions, and Potential Solutions.” Science 320, no. 5878: 889–892. 10.1126/science.1136674. 18487183

[emi70369-bib-0023] Giguere, A. T. , A. E. Taylor , D. D. Myrold , and P. J. Bottomley . 2015. “Nitrification Responses of Soil Ammonia‐Oxidizing Archaea and Bacteria to Ammonium Concentrations.” Soil Science Society of America Journal 79, no. 5: 1366–1374. 10.2136/sssaj2015.03.0107.

[emi70369-bib-0024] Grose, A. L. , S. L. Speir , A. N. Thellman , M. M. Dee , and J. L. Tank . 2022. “Water Transport Pathways Influence the Propagation of Field‐Scale NO^−^‐N Reductions to the Watershed Scale.” Hydrological Processes 36, no. 2: e14476. 10.1002/hyp.14476.

[emi70369-bib-0025] Halvorson, A. D. , C. S. Snyder , A. D. Blaylock , and S. J. Del Grosso . 2014. “Enhanced‐Efficiency Nitrogen Fertilizers: Potential Role in Nitrous Oxide Emission Mitigation.” Agronomy Journal 106, no. 2: 715–722. 10.2134/agronj2013.0081.

[emi70369-bib-0026] Hanrahan, B. R. , J. L. Tank , M. M. Dee , M. T. Trentman , E. M. Berg , and S. K. McMillan . 2018. “Restored Floodplains Enhance Denitrification Compared to Naturalized Floodplains in Agricultural Streams.” Biogeochemistry 141, no. 3: 419–437. 10.1007/s10533-018-0431-4.

[emi70369-bib-0027] Hindmarsh, A. C. , P. N. Brown , K. E. Grant , et al. 2005. “SUNDIALS: Suite of Nonlinear and Differential/Algebraic Equation Solvers.” ACM Transactions on Mathematical Software 31, no. 3: 363–396. 10.1145/1089014.1089020.

[emi70369-bib-0028] Hink, L. , G. W. Nicol , and J. I. Prosser . 2017. “Archaea Produce Lower Yields of N_2_O Than Bacteria During Aerobic Ammonia Oxidation in Soil.” Environmental Microbiology 19, no. 12: 4829–4837. 10.1111/1462-2920.13282. 26971439

[emi70369-bib-0029] Jia, Z. , and R. Conrad . 2009. “ *Bacteria* Rather Than *Archaea* Dominate Microbial Ammonia Oxidation in an Agricultural Soil.” Environmental Microbiology 11, no. 7: 1658–1671. 10.1111/j.1462-2920.2009.01891.x. 19236445

[emi70369-bib-0030] Jiang, H. , L. Huang , Y. Deng , et al. 2014. “Latitudinal Distribution of Ammonia‐Oxidizing Bacteria and Archaea in the Agricultural Soils of Eastern China.” Applied and Environmental Microbiology 80, no. 18: 5593–5602. 10.1128/AEM.01617-14. 25002421 PMC4178612

[emi70369-bib-0031] Jimenez‐Fernandez, O. , M. Schwientek , K. Osenbrück , C. Glaser , C. Schmidt , and J. H. Fleckenstein . 2022. “Groundwater‐Surface Water Exchange as Key Control for Instream and Groundwater Nitrate Concentrations Along a First‐Order Agricultural Stream.” Hydrological Processes 36, no. 2: e14507. 10.1002/hyp.14507.

[emi70369-bib-0032] Joye, S. B. , and J. T. Hollibaugh . 1995. “Influence of Sulfide Inhibition of Nitrification on Nitrogen Regeneration in Sediments.” Science 270, no. 5236: 623–625. 10.1126/science.270.5236.623.

[emi70369-bib-0033] Jung, M.‐Y. , S.‐J. Park , D. Min , et al. 2011. “Enrichment and Characterization of an Autotrophic Ammonia‐Oxidizing Archaeon of Mesophilic Crenarchaeal Group I.1a From an Agricultural Soil.” Applied and Environmental Microbiology 77, no. 24: 8635–8647. 10.1128/AEM.05787-11. 22003023 PMC3233086

[emi70369-bib-0034] Jung, M.‐Y. , C. J. Sedlacek , K. D. Kits , et al. 2021. “Ammonia‐Oxidizing Archaea Possess a Wide Range of Cellular Ammonia Affinities.” ISME Journal 16: 272–283. 10.1038/s41396-021-01064-z. 34316016 PMC8692354

[emi70369-bib-0035] Koch, H. , S. Lücker , M. Albertsen , et al. 2015. “Expanded Metabolic Versatility of Ubiquitous Nitrite‐Oxidizing Bacteria From the Genus *Nitrospira* .” Proceedings of the National Academy of Sciences of the United States of America 112, no. 36: 11371–11376. 10.1073/pnas.1506533112. 26305944 PMC4568715

[emi70369-bib-0036] Koops, H. P. , and A. Pommerening‐Röser . 2001. “Distribution and Ecophysiology of the Nitrifying Bacteria Emphasizing Cultured Species.” FEMS Microbiology Ecology 37, no. 1: 1–9. 10.1016/S0168-6496(01)00137-4.

[emi70369-bib-0037] Krause, S. , C. Tecklenburg , M. Munz , and E. Naden . 2013. “Streambed Nitrogen Cycling Beyond the Hyporheic Zone: Flow Controls on Horizontal Patterns and Depth Distribution of Nitrate and Dissolved Oxygen in the Upwelling Groundwater of a Lowland River.” Journal of Geophysical Research: Biogeosciences 118, no. 1: 54–67. 10.1029/2012JG002122.

[emi70369-bib-0038] Kuypers, M. M. M. , H. K. Marchant , and B. Kartal . 2018. “The Microbial Nitrogen‐Cycling Network.” Nature Reviews Microbiology 16, no. 5: 263–276. 10.1038/nrmicro.2018.9. 29398704

[emi70369-bib-0039] Lagostina, L. , T. Goldhammer , H. Røy , et al. 2015. “Ammonia‐Oxidizing Bacteria of the *Nitrosospira* Cluster 1 Dominate Over Ammonia‐Oxidizing Archaea in Oligotrophic Surface Sediments Near the South Atlantic Gyre.” Environmental Microbiology Reports 7, no. 3: 404–413. 10.1111/1758-2229.12264. 25581373 PMC5008181

[emi70369-bib-0040] Leininger, S. , T. Urich , M. Schloter , et al. 2006. “Archaea Predominate Among Ammonia‐Oxidizing Prokaryotes in Soils.” Nature 442, no. 7104: 806–809. 10.1038/nature04983. 16915287

[emi70369-bib-0041] Levičnik‐Höfferle, Š. , G. W. Nicol , L. Ausec , I. Mandić‐Mulec , and J. I. Prosser . 2012. “Stimulation of Thaumarchaeal Ammonia Oxidation by Ammonia Derived From Organic Nitrogen but Not Added Inorganic Nitrogen.” FEMS Microbiology Ecology 80, no. 1: 114–123. 10.1111/j.1574-6941.2011.01275.x. 22150211

[emi70369-bib-0042] Li, C. , H.‐W. Hu , Q.‐L. Chen , D. Chen , and J.‐Z. He . 2019. “Comammox *Nitrospira* Play an Active Role in Nitrification of Agricultural Soils Amended With Nitrogen Fertilizers.” Soil Biology and Biochemistry 138: 107609. 10.1016/j.soilbio.2019.107609.

[emi70369-bib-0043] Lin, Y. , C. Duan , J. Fan , et al. 2023. “Nitrification Inhibitor 1‐Octyne Inhibits Growth of Comammox Nitrospira but Does Not Alter Their Community Structure in an Acidic Soil.” Journal of Soils and Sediments 23, no. 2: 989–997. 10.1007/s11368-022-03367-w.

[emi70369-bib-0044] Mackin, J. E. , and R. C. Aller . 1984. “Ammonium Adsorption in Marine Sediments.” Limnology and Oceanography 29, no. 2: 250–257. 10.4319/lo.1984.29.2.0250.

[emi70369-bib-0045] Martens‐Habbena, W. , P. M. Berube , H. Urakawa , J. R. de la Torre , and D. A. Stahl . 2009. “Ammonia Oxidation Kinetics Determine Niche Separation of Nitrifying Archaea and Bacteria.” Nature 461, no. 7266, 7266: 976–979. 10.1038/nature08465. 19794413

[emi70369-bib-0046] Mußmann, M. , I. Brito , A. Pitcher , et al. 2011. “Thaumarchaeotes Abundant in Refinery Nitrifying Sludges Express *amoA* but Are Not Obligate Autotrophic Ammonia Oxidizers.” Proceedings of the National Academy of Sciences 108, no. 40: 16771–16776. 10.1073/pnas.1106427108. PMC3189051 21930919

[emi70369-bib-0047] Newbold, J. D. 1992. “Cycles and Spirals of Nutrients.” In The Rivers Handbook: Hydrological and Ecological Principles, edited by P. Calow and G. E. Petts , vol. 1. Blackwell Scientific Publications.

[emi70369-bib-0048] Nogaro, G. , T. Datry , F. Mermillod‐Blondin , S. Descloux , and B. Montuelle . 2010. “Influence of Streambed Sediment Clogging on Microbial Processes in the Hyporheic Zone.” Freshwater Biology 55, no. 6: 1288–1302. 10.1111/j.1365-2427.2009.02352.x.

[emi70369-bib-0049] Norton, J. M. , J. J. Alzerreca , Y. Suwa , and M. G. Klotz . 2002. “Diversity of Ammonia Monooxygenase Operon in Autotrophic Ammonia‐Oxidizing Bacteria.” Archives of Microbiology 177, no. 2: 139–149. 10.1007/s00203-001-0369-z. 11807563

[emi70369-bib-0050] Pei, A. Y. , W. E. Oberdorf , C. W. Nossa , et al. 2010. “Diversity of 16S rRNA Genes Within Individual Prokaryotic Genomes.” Applied and Environmental Microbiology 76, no. 12: 3886–3897. 10.1128/AEM.02953-09. 20418441 PMC2893482

[emi70369-bib-0051] Peterson, B. J. , W. M. Wollheim , P. J. Mulholland , et al. 2001. “Control of Nitrogen Export From Watersheds by Headwater Streams.” Science 292, no. 5514: 86–90. 10.1126/science.1056874. 11292868

[emi70369-bib-0052] Prosser, J. I. 2012. “Ecosystem Processes and Interactions in a Morass of Diversity.” FEMS Microbiology Ecology 81, no. 3: 507–519. 10.1111/j.1574-6941.2012.01435.x. 22715974

[emi70369-bib-0053] Ramanathan, B. , A. M. Boddicker , T. M. Roane , and A. C. Mosier . 2017. “Nitrifier Gene Abundance and Diversity in Sediments Impacted by Acid Mine Drainage.” Frontiers in Microbiology 8, no. November: 2136. 10.3389/fmicb.2017.02136. 29209281 PMC5701628

[emi70369-bib-0054] Seyboldt, A. 2021. Sunode. V. 0.2.2. Zenodo. 10.5281/zenodo.5213947.

[emi70369-bib-0055] Sonthiphand, P. , and J. Neufeld . 2014. “Nitrifying Bacteria Mediate Aerobic Ammonia Oxidation and Urea Hydrolysis Within the Grand River.” Aquatic Microbial Ecology 73, no. 2: 151–162. 10.3354/ame01712.

[emi70369-bib-0056] Spasov, E. , J. M. Tsuji , L. A. Hug , et al. 2020. “High Functional Diversity Among Nitrospira Populations That Dominate Rotating Biological Contactor Microbial Communities in a Municipal Wastewater Treatment Plant.” ISME Journal 14, no. 7: 1857–1872. 10.1038/s41396-020-0650-2. 32332864 PMC7305129

[emi70369-bib-0057] Stein, L. Y. 2014. “Heterotrophic Nitrification and Nitrifier Denitrification.” In Nitrification, edited by B. B. Ward , D. J. Arp , and M. G. Klotz , 95–114. ASM Press. 10.1128/9781555817145.ch5.

[emi70369-bib-0058] Storey, R. G. , D. D. Williams , and R. R. Fulthorpe . 2004. “Nitrogen Processing in the Hyporheic Zone of a Pastoral Stream.” Biogeochemistry 69, no. 3: 285–313. 10.1023/B:BIOG.0000031049.95805.ec.

[emi70369-bib-0059] Störiko, A. , Z. Wang , A. Jung , et al. 2025. Bayesian Simulation of Ammonium Oxidation in Microcosm Experiments: Modeling Code and Data. Version v2.0.0. Zenodo. 10.5281/zenodo.17087314.

[emi70369-bib-0060] Strauss, E. A. , and G. A. Lamberti . 2002. “Effect of Dissolved Organic Carbon Quality on Microbial Decomposition and Nitrification Rates in Stream Sediments.” Freshwater Biology 47, no. 1: 65–74. 10.1046/j.1365-2427.2002.00776.x.

[emi70369-bib-0061] Taylor, A. E. , N. Vajrala , A. T. Giguere , et al. 2013. “Use of Aliphatic *n*‐Alkynes to Discriminate Soil Nitrification Activities of Ammonia‐Oxidizing Thaumarchaea and Bacteria.” Applied and Environmental Microbiology 79, no. 21: 6544–6551. 10.1128/AEM.01928-13. 23956393 PMC3811497

[emi70369-bib-0062] Tourna, M. , M. Stieglmeier , A. Spang , et al. 2011. “ *Nitrososphaera viennensis*, an Ammonia Oxidizing Archaeon From Soil.” Proceedings of the National Academy of Sciences of the United States of America 108, no. 20: 8420–8425. 10.1073/pnas.1013488108. 21525411 PMC3100973

[emi70369-bib-0063] Triska, F. J. , J. H. Duff , and R. J. Avanzino . 1993. “The Role of Water Exchange Between a Stream Channel and Its Hyporheic Zone in Nitrogen Cycling at the Terrestrial‐Aquatic Interface.” Hydrobiologia 251, no. 1–3: 167–184. 10.1007/BF00007177.

[emi70369-bib-0064] Van Kessel, M. A. H. J. , D. R. Speth , M. Albertsen , et al. 2015. “Complete Nitrification by a Single Microorganism.” Nature 528, no. 7583: 555–559. 10.1038/nature16459. 26610025 PMC4878690

[emi70369-bib-0065] Vehtari, A. , A. Gelman , D. Simpson , B. Carpenter , and P.‐C. Bürkner . 2021. “Rank‐Normalization, Folding, and Localization: An Improved $\hat{R}$ for Assessing Convergence of MCMC.” Bayesian Analysis 16, no. 2: 667–718. 10.1214/20-BA1221.

[emi70369-bib-0066] Verhamme, D. T. , J. I. Prosser , and G. W. Nicol . 2011. “Ammonia Concentration Determines Differential Growth of Ammonia‐Oxidising Archaea and Bacteria in Soil Microcosms.” ISME Journal 5, no. 6: 1067–1071. 10.1038/ismej.2010.191. 21228892 PMC3131854

[emi70369-bib-0067] Wagner, M. , G. Rath , H.‐P. Koops , J. Flood , and R. Amann . 1996. “In Situ Analysis of Nitrifying Bacteria in Sewage Treatment Plants.” Water Science and Technology 34, no. 1–2: 237–244. 10.1016/0273-1223(96)00514-8.

[emi70369-bib-0068] Wang, Z. 2023. Microbial Nitrogen Cycling in Sediments of an Agricultural Stream as Impacted by Stream‐Groundwater Exchange. Technische Universität München. https://mediatum.ub.tum.de/1704068.

[emi70369-bib-0069] Wang, Z. , O. Jimenez‐Fernandez , K. Osenbrück , et al. 2022. “Streambed Microbial Communities in the Transition Zone Between Groundwater and a First‐Order Stream as Impacted by Bidirectional Water Exchange.” Water Research 217, no. June: 118334. 10.1016/j.watres.2022.118334. 35397370

[emi70369-bib-0070] Wright, C. L. , and L. E. Lehtovirta‐Morley . 2023. “Nitrification and Beyond: Metabolic Versatility of Ammonia Oxidising Archaea.” ISME Journal 17, no. 9, 9: 1358–1368. 10.1038/s41396-023-01467-0. 37452095 PMC10432482

[emi70369-bib-0071] Xia, F. , J. G. Wang , T. Zhu , B. Zou , S. K. Rhee , and Z. X. Quan . 2018. “Ubiquity and Diversity of Complete Ammonia Oxidizers (Comammox).” Applied and Environmental Microbiology 84, no. 24: e01390‐18. 10.1128/AEM.01390-18. 30315079 PMC6275355

[emi70369-bib-0072] Xia, S. , Y. Shi , Y. Fu , and X. Ma . 2005. “DGGE Analysis of 16S rDNA of Ammonia‐Oxidizing Bacteria in Chemical‐Biological Flocculation and Chemical Coagulation Systems.” Applied Microbiology and Biotechnology 69, no. 1: 99–105. 10.1007/s00253-005-0035-5. 15983805

[emi70369-bib-0073] Yang, Y. , J. Zhang , Q. Zhao , et al. 2016. “Sediment Ammonia‐Oxidizing Microorganisms in Two Plateau Freshwater Lakes at Different Trophic States.” Microbial Ecology 71, no. 2: 257–265. 10.1007/s00248-015-0642-3. 26111964

[emi70369-bib-0074] Zhang, L. , Y. Guan , and S. C. Jiang . 2021. “Investigations of Soil Autotrophic Ammonia Oxidizers in Farmlands Through Genetics and Big Data Analysis.” Science of the Total Environment 777: 146091. 10.1016/j.scitotenv.2021.146091.

[emi70369-bib-0075] Zhang, S. , W. Qin , X. Xia , et al. 2020. “Ammonia Oxidizers in River Sediments of the Qinghai‐Tibet Plateau and Their Adaptations to High‐Elevation Conditions.” Water Research 173: 115589. 10.1016/j.watres.2020.115589. 32058148

[emi70369-bib-0076] Zhang, Y. , X. Xie , N. Jiao , S. S. Y. Hsiao , and S. J. Kao . 2014. “Diversity and Distribution of *amoA*‐Type Nitrifying and *nirS*‐Type Denitrifying Microbial Communities in the Yangtze River Estuary.” Biogeosciences 11, no. 8: 2131–2145. 10.5194/bg-11-2131-2014.

[emi70369-bib-0077] Zheng, Y. , B. Wang , P. Gao , et al. 2024. “Novel Order‐Level Lineage of Ammonia‐Oxidizing Archaea Widespread in Marine and Terrestrial Environments.” ISME Journal 18, no. 1: wrad002. 10.1093/ismejo/wrad002. 38365232 PMC10811736

[emi70369-bib-0078] Zhu, T. , T. Meng , J. Zhang , W. Zhong , C. Müller , and Z. Cai . 2015. “Fungi‐Dominant Heterotrophic Nitrification in a Subtropical Forest Soil of China.” Journal of Soils and Sediments 15, no. 3: 705–709. 10.1007/s11368-014-1048-4.

