# Supplementary material for: Linking Abundance and Activity of Ammonia‐Oxidising Bacteria and Archaea in an Agriculturally Impacted First‐Order Stream
Source: Environ Microbiol. 2026 Jul 13;28(7):e70369. doi: 10.1111/1462-2920.70369 (PMC13365662; doi:10.1111/1462-2920.70369)
Supplement: Supplementary file 1 — Table S1: Schönbrunnen stream discharge (Q), groundwater table depth d (in metres below ground level) and major water chemistry parameters in both the stream water and adjacent groundwater measured in the sampling month (June 2020). Table S2: Simulated stream water medium modified from a previous study (de la Torre 2008). Table S3: Primers and annealing temperatures (T) for qPCR amplification of bacterial and archaeal 16S rRNA genes and amoA genes. Table S4: Bacterial and archaeal 16S rRNA gene copy numbers per gramme wet sediment before (Day 0) and after (Day 14) the microcosm incubation as determined by quantitative PCR. Table S5: Bacterial and archaeal amoA gene copy numbers per gramme wet sediment before (Day 0) and after (Day 14) the microcosm incubation as determined by quantitative PCR. Figure S1: The in situ Shannon diversity index (H′) in all sediment samples. Figure S2: Relative abundance of Top 20 most abundant microbial lineages at the family level detected in Schönbrunnen sediments prior to the microcosm incubation (T 0). Figure S3: Relative abundance of Nitrosomonadaceae‐affiliated ASVs clustered at the genus level before and after the microcosm incubation. Figure S4: Increase of amoA gene concentrations from Days 0 to 14. Figure S5: Ammonium oxidation rates calculated with an ammonium concentration of 10 μM, using the amoA concentrations measured in the in situ sediment samples the posterior samples of kinetic parameters. Figure S6: Posterior distributions of model parameters describing the oxidation of ammonium and growth of AOA and AOB for microcosms from different stream segments and depths. Table S6: Model parameters of the reaction model and the corresponding prior distributions for the Bayesian estimation of parameter values. Table S7: Parameters for the reaction model of the microcosm experiments that were set to fixed values. Figure S7: Measured and simulated time series of ammonium, nitrate and bacterial and archaeal amoA gene concentrations [file EMI-28-e70369-s001.pdf]

SUPPORTING INFORMATION FOR

"Linking abundance and activity of ammonia-oxidizing bacteria and archaea in an agriculturally impacted first-order stream"

Zhe Wang[1,2,#*], Anna Störiko[3,4,#*], Aileen Jung[1], Daniel Straub[3,5], Olaf A. Cirpka[3], Holger Pagel[6,7,8], Tillmann Lueders[1]

[1] Chair of Ecological Microbiology, Bayreuth Center of Ecology and Environmental Research (BayCEER), University of Bayreuth, Bayreuth, Germany
[2] Department of Earth and Planetary Sciences, ETH Zürich, Zürich, Switzerland
[3] Department of Geosciences, University of Tübingen, Tübingen, Germany
[4] Department of Water Management, Delft University of Technology, Delft, The Netherlands
[5] Quantitative Biology Center, University of Tübingen, Tübingen, Germany
[6] Department of Biogeophysics, Institute of Soil Science and Land Evaluation, University of Hohenheim, Stuttgart, Germany
[7] Institute of Bio- and Geosciences IBG-3: Agrosphere, Forschungszentrum Jülich GmbH, Jülich, Germany
[8] Institute of Crop Science and Resource Conservation, University of Bonn, Bonn, Germany
[#] These authors made an equal contribution to the study.
[*] Corresponding author. E-mail address: zhe.wang@eaps.ethz.ch, a.storiko@tudelft.nl

Contents

1 Supplementary Materials and Methods 2
1.1 DNA isolation and amplicon sequencing 2
1.2 qPCR measurements 2
1.3 Sequencing data analysis 3
1.4 Bacterial *amoA* gene amplicon sequences analysis 3

2 Supplementary Tables 5

3 Supplementary Figures 9

4 Bayesian Parameter Estimation for the Reaction Model 13
4.1 Prior distributions 13
4.2 Preprocessing of measurement data 13
4.3 Sampler convergence 14
4.4 Posterior distributions 14

5 Simulated Concentration Time Series 17

6 Supplementary References 20

1 Supplementary Materials and Methods

1.1 DNA isolation and amplicon sequencing

Isolation of genomic DNA from preprocessed sediment samples was conducted according to a protocol published previously with minor modifications as the extraction process was conducted at 4 °C rather than 20 °C from the start to the end (Pilloni et al., 2012). Extracted DNA was then quantified with a Qubit Fluorometer (Thermo Fisher Scientific, Waltham, MA). Amplicons of the V4 region of prokaryotic 16S ribosomal RNA (rRNA) genes were amplified using the NEBNext® High-Fidelity 2X PCR Master Mix (New England Biolabs GmbH, Frankfurt am Main, Germany), and universal Illumina-adaptor primers 515f (5′-GTGYCAGCMGCCGCGGTAA-3′) and 806Rn (5′-GGACTACNVGGGTWTCTAAT-3′) (Apprill et al., 2015; Caporaso et al., 2011; Parada et al., 2016). Bacterial *amoA* genes were amplified using 1 x Brilliant III Ultra-Fast qPCR Master Mix (Agilent, Santa Clara, CA, USA), and adaptor primers amoA-1F (5′-GGGGTTTCTACTGGTGGT-3′) and amoA-2R (5′-CCCCTCKGSAAAGCCTTCTTC-3′) (Rotthauwe et al., 1997). 16S rRNA and *amoA* gene amplicons from duplicate DNA extracts (always replicate a and replicate b from each sampling spot) were pooled before sequencing, as all three microcosms replicates followed similar dynamics in nitrate concentrations during incubation. Further library preparation (Nextera, Illumina) and sequencing on an Illumina MiSeq platform (Illumina, San Diego, CA, United States) using 250 bp paired-end with v2 chemistry was performed by Microsynth AG, Switzerland.

1.2 qPCR measurements

The quantification of prokaryotic 16S rRNA genes and *amoA* was performed on a real-time polymerase chain reaction (PCR) system CFX96 (Biorad, Feldkirchen, Germany) and calibrated using standard curves from serially diluted synthetic gene fragments of known concentration (gBlocks, Integrated DNA Technologies Leuven, Belgium). For bacterial 16S rRNA genes, a 980 bp-fragment of the *Aromatoleum toluolicum* T^T(AF12946) was used. A 980 bp-fragment from *Methanosarcina barkeri* DSM 800 (NR_025303) was used for Archaeal 16S rRNA genes. For bacterial *amoA* genes, a 730 bp-fragment of the *Nitrosomonas europaea* ATCC 19178 (JN099309.1) was used. A 874 bp-fragment from Candidatus Nitrosotenuis cloacae SAT1 (CP011097) was used for archaeal *amoA* genes.

Each standard curve reached an R^2-value greater than 0.99. All replicate samples for quantitative PCR (qPCR) analyses were quantified in technical duplicates, and the reaction efficiency was between 80 % and 92 % for all target genes. Reactions were performed in a total volume of 40 µL, containing 20 µL Brilliant III Ultra-Fast SYBR Green qPCR Master Mix (Agilent, Santa Clara, CA, USA), 0.25 µL 50 µM primer, 0.4 µL 20 µg μL^{-1} BSA (Roche, Rotkreuz, Switzerland) and 2 µL DNA template. The primers

used to amplify the target genes are shown in table S3. qPCR thermal profiles were as follows: initial denaturation at 95 °C (3 min), 35–40 cycles of denaturation at 95 °C (30 s), annealing at a given temperature (30 s), elongation at 72 °C (30 s), followed by a final melting at 95 °C (30 s), and a melting curve recorded between 55 °C and 95 °C. The annealing temperature of the primer was set to 52 °C for bacterial 16S rRNA genes, 56 °C for archaeal 16S rRNA genes, 56 °C for bacterial *amoA* genes, and 57 °C for archaeal *amoA* genes.

1.3 Sequencing data analysis

Raw sequencing data of bacteria/archaeal 16S rRNA gene amplicons were first processed using Cutadapt (version 1.14) (Martin, 2011) to trim primers. Trimmed adapter and primer-free sequences were then processed using the DADA2 package (version 1.16.0) (Callahan et al., 2016) in R (version 4.0.3) (R Core Team, 2019) to merge forward and reverse reads, infer the exact amplicon sequencing variants (ASVs), and remove PCR chimeras. In total, 10 399 ASV sequences were imported into the IDTAXA Classifier in package DECIPHER (version 2.18.1) (Wright, 2016), using a confidence level of 50 % (high), to map ASV sequences against the SILVA SSU database (release 138) for taxonomic classification (Quast et al., 2012). ASVs classified as "mitochondria", "chloroplasts", or "unclassified root" were removed. In addition, ASVs with an abundance <0.0001 % across all samples were removed. Total reads were rarefied to 19 400 reads per sample, which was the lowest number of reads observed among all samples, without sacrificing fully observed richness. The final cleaned ASV table contained 9589 ASVs and further analyzed for the alpha and beta diversity and visualized mainly with the phyloseq (version 1.34.0), ggplot2 (version 3.3.2), and vegan (version 2.5-6) packages (McMurdie & Holmes, 2013; Oksanen et al., 2019; Wickham, 2016) in R.

1.4 Bacterial *amoA* gene amplicon sequences analysis

Raw sequencing data of bacteria *amoA* gene amplicons were processed with nf-core/ampliseq v2.0.0 (Ewels et al., 2020; Straub et al., 2020) using Nextflow v21.03.0.edge (Di Tommaso et al., 2017) and singularity v3.4.2 (Kurtzer et al., 2017). Processed bacterial *amoA* sequences were grouped into operational taxonomic units (OTUs) with a 95 % similarity threshold in R version 3.6.1 (R Core Team, 2019) with DECIPHER v2.14.0 (Wright, 2016). Primers were trimmed, and untrimmed sequences were discarded with Cutadapt version 3.2 (Martin, 2011). Adapter and primer-free sequences were processed with DADA2 v1.18.0 (Callahan et al., 2016) to eliminate PhiX contamination, trim reads (before median quality drops below 25; forward reads were trimmed at 232 bp and reverse reads at 229 bp), correct errors, merge read pairs (adjusted to minOverlap = 7, maxMismatch = 0; minOverlap = 5 yielded only one additional ASV with 53 reads in

only one sample, we chose to disregard this ASV and continue with minOverlap = 7), and remove PCR chimeras. At this point, 11 972 ASVs were obtained across all samples. Next, bacterial *amoA* ASVs were then further grouped into OTUs with a 95 % similarity threshold in R version 3.6.1 (R Core Team, 2019) with DECIPHER v2.14.0 (Wright, 2016). 764 of 1104 OTU sequences were exactly 452 bp long, 340 OTUs with other lengths were filtered out because they were not *amoA* sequences according to BLASTn (Altschul et al., 1990). The final OTU table was rarefied to 46 570 reads per sample, which was the lowest number of reads observed in a single sample.

The rarefied OTU tables were further used to analyze the alpha and beta diversity and visualized mainly with the phyloseq (version 1.34.0), ggplot2 (version 3.3.2), and vegan (version 2.5-6) packages (McMurdie & Holmes, 2013; Oksanen et al., 2019; Wickham, 2016) in R. The FastTree (Price et al., 2009) algorithm was applied for generating a midpoint-rooted phylogenetic tree after OTU sequence alignment by the DECIPHER package (Wright, 2016, version 2.12.0). Maximum likelihood phylogenetic trees were constructed for *amoA* OTUs with MEGA-X (S. Kumar et al., 2018), aligned with selected reference bacterial *amoA* gene sequences from NCBI databases (NCBI Resource Coordinators, 2018), using the ClustalW algorithm with default settings (1000 bootstrap replications).

Differential abundance analyses were performed using unrarefied reads counts (McMurdie & Holmes, 2014) using the DESeq2 package (Love et al., 2014, version 1.30.1). For each OTU, DESeq2 fits a negative binomial generalized linear model accounting for differences in library size. Wald tests were applied to evaluate whether $\log_2$-fold changes between conditions differed significantly from zero, and resulting *p*-values were adjusted for multiple testing (Benjamini & Hochberg, 1995).

2 Supplementary Tables

Table S1: Schönbrunnen stream discharge (Q), groundwater table depth d (in meters below ground level) and major water chemistry parameters in both the stream water and adjacent groundwater measured in the sampling month (June 2020).

Sampling location	Q [$L\,s^{-1}$]	d [m]	EC [$\mu S\,cm^{-1}$]	Ca^{2+} [mM]	NH_4^+ [mM]	NO_3^- [mM]	Cl^- [mM]	SO_4^{2-} [mM]
Stream Water								
Up			1053	4.05	0	0.84	0.46	1.63
Mid		0.37	954	3.18	0	0.91	0.46	1.76
Down			989	3.78	0	0.64	0.43	1.88
Groundwater								
GWS 02		0.9	1049	4.03	0	0.89	0.46	1.69
GWS 08		1.1	2600	16.08	5.5	0.01	0.43	11.85
GWS 12		1.6	941	3.73	0	0.54	0.43	0.02
GWS 19		0.6	849	3.05	0	0.58	0.36	0.51
GWS 23		0.4	1057	4.38	0.01	0.02	0.37	1.68
GWS 26		0.6	1380	6.85	0.03	0	0.29	4.07

Table S2: Simulated stream water medium modified from a previous study (de la Torre et al., 2008).

Component	Amount
NaCl	1.0 g
$MgCl \cdot 6\,H_2O$	0.4 g
$CaCl_2 \cdot 2\,H_2O$	0.1 g
KCl	0.5 g
$NaHCO_3$	0.4 g
Vitamin solution	1.0 mL
Trace element solution	1.0 mL
Selenite-tungstate solution	1.0 mL
Phosphate buffer	1.0 mL
ddH_2O	Add to total 1000 mL (pH 7.0)

Table S3: Primers and annealing temperatures (T) for qPCR amplification of bacterial and archaeal 16S rRNA genes and *amoA* genes.

Genes	Primers	Sequence (5′–3′)	T	Reference
Bacterial 16S rRNA genes	Ba519f<br>Ba907r	CAGCMGCCGCGGTAATA<br>CCGTCAATTCCTTTGAGTTT	52 °C	Lane (1991)
Archaeal 16S rRNA genes	Ar109f<br>Ar912rt	ACKGCTCAGTAACACGT<br>GTGCTCCCCCGCCAATTCCTTTA	56 °C	Lueders et al. (2004)
Bacterial *amoA* genes	amoA-1F<br>amoA-2R	GGGGTTTCTACTGGTGGT<br>CCCCTCKGSAAAGCCTTCTTC	57 °C	Rotthauwe et al. (1997)
Archaeal *amoA* genes	Arch-amoAF<br>Arch-amoAR	STAATGGTCTGGCTTAGACG<br>GCGGCCATCCATCTGTATGT	56 °C	Francis et al. (2005)

Table S4: Bacterial and archaeal 16S rRNA gene copy numbers per gram wet sediment before (day 0) and after (day 14) the microcosm incubation as determined by quantitative PCR. qPCR results are shown as the mean value and standard deviation (STD) from replicate microcosms ($n = 2$ for pre-incubation data; $n = 3$ for post-incubation data) and technical replicates ($n = 2$) of each microcosm. A machine-readable version of the data (.xlsx file) is available in Störiko et al. (2025).

			bacterial 16S rRNA genes				archaeal 16S rRNA genes			
			day 0		day 14		day 0		day 14	
			Mean	STD	Mean	STD	Mean	STD	Mean	STD
section	depth	treatment								
Up	15 cm	Ammonium + 1-octyne	1.97×10^8	1.48×10^7	3.51×10^8	8.77×10^7	5.51×10^7	$n < 3$	5.23×10^7	1.48×10^7
Up	15 cm	Ammonium + acetylene	1.32×10^8	6.99×10^7	3.58×10^8	8.61×10^7	3.54×10^7	1.61×10^7	5.92×10^7	1.72×10^7
Up	15 cm	Ammonium + no inhibitor	1.67×10^8	4.46×10^7	3.71×10^8	5.97×10^7	5.61×10^7	$n < 3$	7.20×10^7	1.02×10^7
Up	15 cm	no Ammonium + no inhibitor	7.53×10^7	5.59×10^7	2.97×10^8	1.11×10^8	2.20×10^7	1.58×10^7	5.93×10^7	2.32×10^7
Up	5 cm	Ammonium + 1-octyne	3.26×10^8	8.22×10^7	4.18×10^8	8.82×10^7	3.91×10^7	1.28×10^7	4.83×10^7	1.77×10^7
Up	5 cm	Ammonium + acetylene	2.40×10^8	1.04×10^7	3.65×10^8	4.33×10^7	3.63×10^7	5.33×10^6	4.19×10^7	7.19×10^6
Up	5 cm	Ammonium + no inhibitor	2.55×10^8	5.89×10^7	3.25×10^8	1.21×10^7	2.56×10^7	6.68×10^6	5.09×10^7	8.70×10^6
Up	5 cm	no Ammonium + no inhibitor	1.75×10^8	1.30×10^7	4.72×10^8	4.12×10^7	2.72×10^7	6.99×10^5	6.21×10^7	6.70×10^6
Mid	15 cm	Ammonium + 1-octyne	1.94×10^8	1.36×10^7	4.64×10^8	1.41×10^8	1.84×10^7	1.68×10^6	3.32×10^7	7.94×10^6
Mid	15 cm	Ammonium + acetylene	2.42×10^8	6.05×10^6	3.37×10^8	5.57×10^7	2.55×10^7	1.21×10^5	1.99×10^7	1.85×10^6
Mid	15 cm	Ammonium + no inhibitor	1.80×10^8	2.65×10^7	4.14×10^8	4.89×10^7	1.52×10^7	1.74×10^6	2.61×10^7	3.68×10^6
Mid	15 cm	no Ammonium + no inhibitor	1.38×10^8	4.16×10^7	4.15×10^8	5.07×10^7	1.07×10^7	3.49×10^6	2.65×10^7	2.41×10^6
Mid	5 cm	Ammonium + 1-octyne	2.84×10^8	4.10×10^6	1.03×10^9	1.17×10^8	8.36×10^7	6.79×10^6	1.98×10^8	3.15×10^7
Mid	5 cm	Ammonium + acetylene	2.83×10^8	1.32×10^8	8.66×10^8	9.84×10^7	7.85×10^7	3.73×10^7	1.76×10^8	3.26×10^7
Mid	5 cm	Ammonium + no inhibitor	4.18×10^8	2.28×10^7	8.99×10^8	4.31×10^7	1.14×10^8	3.96×10^6	2.35×10^8	2.00×10^7
Mid	5 cm	no Ammonium + no inhibitor	3.25×10^8	6.19×10^7	6.91×10^8	5.48×10^7	9.10×10^7	1.51×10^7	1.52×10^8	8.07×10^7
Down	15 cm	Ammonium + 1-octyne	2.06×10^8	7.77×10^6	5.71×10^8	4.88×10^7	1.85×10^7	2.98×10^5	2.25×10^7	3.78×10^6
Down	15 cm	Ammonium + acetylene	2.12×10^8	6.40×10^7	6.79×10^8	2.94×10^7	1.36×10^7	5.65×10^6	3.22×10^7	4.90×10^6
Down	15 cm	Ammonium + no inhibitor	1.73×10^8	3.76×10^7	6.14×10^8	6.63×10^7	1.40×10^7	2.03×10^6	3.21×10^7	1.83×10^6
Down	15 cm	no ammonium + no inhibitor	2.12×10^8	5.24×10^6	5.67×10^8	9.61×10^7	1.73×10^7	2.66×10^5	2.91×10^7	3.50×10^6
Down	5 cm	Ammonium + 1-octyne	1.68×10^8	2.21×10^7	6.03×10^8	5.87×10^7	1.37×10^7	2.38×10^6	4.57×10^7	1.98×10^6
Down	5 cm	Ammonium + acetylene	2.08×10^8	5.95×10^7	5.86×10^8	1.46×10^8	2.25×10^7	6.10×10^6	3.21×10^7	6.32×10^6
Down	5 cm	Ammonium + no inhibitor	1.99×10^8	3.18×10^7	5.11×10^8	1.35×10^8	1.81×10^7	2.61×10^6	3.79×10^7	1.21×10^7
Down	5 cm	no Ammonium + no inhibitor	2.25×10^8	1.86×10^7	4.96×10^8	2.65×10^7	2.17×10^7	1.45×10^6	3.55×10^7	1.83×10^6

Table S5: Bacterial and archaeal *amoA* gene copy numbers per gram wet sediment before (day 0) and after (day 14) the microcosm incubation as determined by quantitative PCR. qPCR results are shown as the mean value and standard deviation (STD) from replicate microcosms ($n = 2$ for pre-incubation data; $n = 3$ for post-incubation data) and technical replicates ($n = 2$) of each microcosm. A machine-readable version of the data (.xlsx file) is available in Störiko et al. (2025).

			bacterial *amoA* genes				archaeal *amoA* genes			
			day 0		day 14		day 0		day 14	
			Mean	STD	Mean	STD	Mean	STD	Mean	STD
section	depth	treatment								
Up	15 cm	Ammonium + 1-octyne	5.78×10^{4}	$n < 3$	4.74×10^{5}	2.29×10^{5}	1.15×10^{6}	$n < 3$	3.09×10^{6}	9.92×10^{5}
Up	15 cm	Ammonium + acetylene	4.71×10^{4}	1.62×10^{4}	1.07×10^{5}	2.67×10^{4}	6.37×10^{5}	2.85×10^{5}	3.70×10^{6}	1.19×10^{6}
Up	15 cm	Ammonium + no inhibitor	3.03×10^{4}	1.92×10^{3}	3.55×10^{6}	6.30×10^{5}	8.66×10^{5}	$n < 3$	5.52×10^{6}	1.10×10^{6}
Up	15 cm	no Ammonium + no inhibitor	2.15×10^{4}	1.00×10^{4}	9.80×10^{4}	1.60×10^{4}	3.50×10^{5}	2.82×10^{5}	3.99×10^{6}	1.57×10^{6}
Up	5 cm	Ammonium + 1-octyne	7.64×10^{4}	3.29×10^{4}	1.66×10^{6}	5.30×10^{5}	2.70×10^{6}	1.01×10^{6}	1.88×10^{7}	8.95×10^{6}
Up	5 cm	Ammonium + acetylene	6.18×10^{4}	6.78×10^{3}	1.09×10^{5}	1.83×10^{4}	1.85×10^{6}	1.70×10^{5}	8.04×10^{6}	1.75×10^{6}
Up	5 cm	Ammonium + no inhibitor	5.22×10^{4}	1.00×10^{4}	3.21×10^{6}	7.31×10^{5}	2.48×10^{6}	7.60×10^{3}	1.81×10^{7}	2.77×10^{6}
Up	5 cm	no Ammonium + no inhibitor	5.42×10^{4}	5.85×10^{3}	1.68×10^{5}	2.56×10^{4}	1.48×10^{6}	4.94×10^{4}	1.91×10^{7}	2.72×10^{6}
Mid	15 cm	Ammonium + 1-octyne	4.62×10^{4}	4.83×10^{3}	2.03×10^{5}	4.09×10^{4}	4.00×10^{5}	9.80×10^{4}	1.43×10^{6}	5.11×10^{5}
Mid	15 cm	Ammonium + acetylene	4.92×10^{4}	2.42×10^{3}	7.05×10^{4}	1.07×10^{4}	6.76×10^{5}	4.42×10^{3}	7.24×10^{5}	7.37×10^{4}
Mid	15 cm	Ammonium + no inhibitor	3.16×10^{4}	1.74×10^{3}	3.72×10^{6}	5.13×10^{5}	2.49×10^{5}	1.92×10^{3}	1.19×10^{6}	1.84×10^{5}
Mid	15 cm	no Ammonium + no inhibitor	3.05×10^{4}	9.21×10^{3}	1.79×10^{5}	3.24×10^{4}	1.75×10^{5}	5.64×10^{4}	1.11×10^{6}	1.12×10^{5}
Mid	5 cm	Ammonium + 1-octyne	3.98×10^{5}	4.05×10^{4}	6.37×10^{6}	3.21×10^{6}	2.63×10^{6}	1.09×10^{6}	1.04×10^{7}	3.72×10^{6}
Mid	5 cm	Ammonium + acetylene	3.63×10^{5}	2.11×10^{5}	1.14×10^{6}	3.74×10^{6}	2.06×10^{6}	9.04×10^{5}	9.19×10^{6}	2.82×10^{6}
Mid	5 cm	Ammonium + no inhibitor	3.68×10^{5}	7.62×10^{4}	1.04×10^{7}	5.55×10^{6}	2.10×10^{6}	3.30×10^{4}	1.18×10^{7}	3.18×10^{6}
Mid	5 cm	no Ammonium + no inhibitor	2.99×10^{5}	2.85×10^{4}	8.47×10^{5}	5.23×10^{5}	1.64×10^{6}	3.58×10^{5}	5.93×10^{6}	3.28×10^{6}
Down	15 cm	Ammonium + 1-octyne	3.10×10^{4}	4.40×10^{3}	1.31×10^{5}	3.55×10^{4}	1.09×10^{6}	4.74×10^{4}	2.74×10^{6}	2.80×10^{5}
Down	15 cm	Ammonium + acetylene	3.21×10^{4}	2.82×10^{3}	1.09×10^{5}	9.67×10^{3}	8.48×10^{5}	6.96×10^{3}	3.33×10^{6}	6.03×10^{5}
Down	15 cm	Ammonium + no inhibitor	3.58×10^{4}	1.25×10^{3}	1.37×10^{6}	6.07×10^{5}	8.48×10^{5}	2.38×10^{5}	2.99×10^{6}	2.08×10^{5}
Down	15 cm	no ammonium + no inhibitor	3.66×10^{4}	2.80×10^{3}	1.11×10^{5}	1.10×10^{4}	9.99×10^{5}	3.58×10^{4}	2.51×10^{6}	3.56×10^{5}
Down	5 cm	Ammonium + 1-octyne	8.84×10^{4}	7.07×10^{3}	1.70×10^{6}	2.84×10^{5}	1.25×10^{6}	1.73×10^{5}	5.38×10^{6}	4.65×10^{5}
Down	5 cm	Ammonium + acetylene	9.88×10^{4}	1.30×10^{4}	2.75×10^{5}	6.62×10^{4}	1.03×10^{6}	2.98×10^{5}	3.00×10^{6}	9.95×10^{5}
Down	5 cm	Ammonium + no inhibitor	9.43×10^{4}	2.62×10^{3}	2.02×10^{6}	7.50×10^{5}	5.59×10^{5}	1.61×10^{5}	3.75×10^{6}	2.25×10^{6}
Down	5 cm	no Ammonium + no inhibitor	1.18×10^{5}	2.02×10^{4}	1.95×10^{5}	3.18×10^{4}	6.93×10^{5}	7.14×10^{4}	3.41×10^{6}	2.19×10^{5}

3 Supplementary Figures

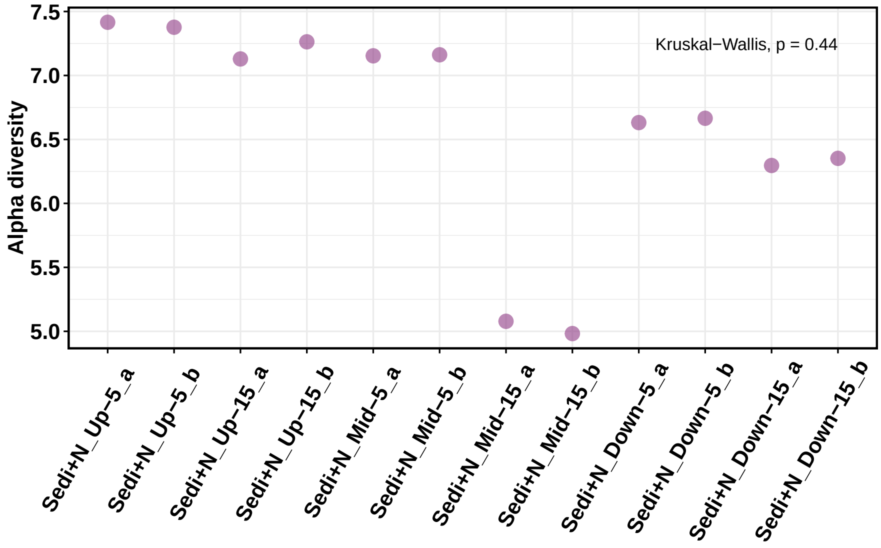

Figure S1: The *in-situ* Shannon diversity index (H') in all sediment samples. "Up", "Mid", and "Down" in the sample names on x-axis indicate sampling location upstream, midstream, and downstream, respectively. The sample name suffix ("a" and "b") indicates biological duplicates. Differences in (H') between groups were tested for statistical significance using Kruskal-Wallis followed by Dunn's post-hoc test with Bonferroni correction ($p = 0.44$).

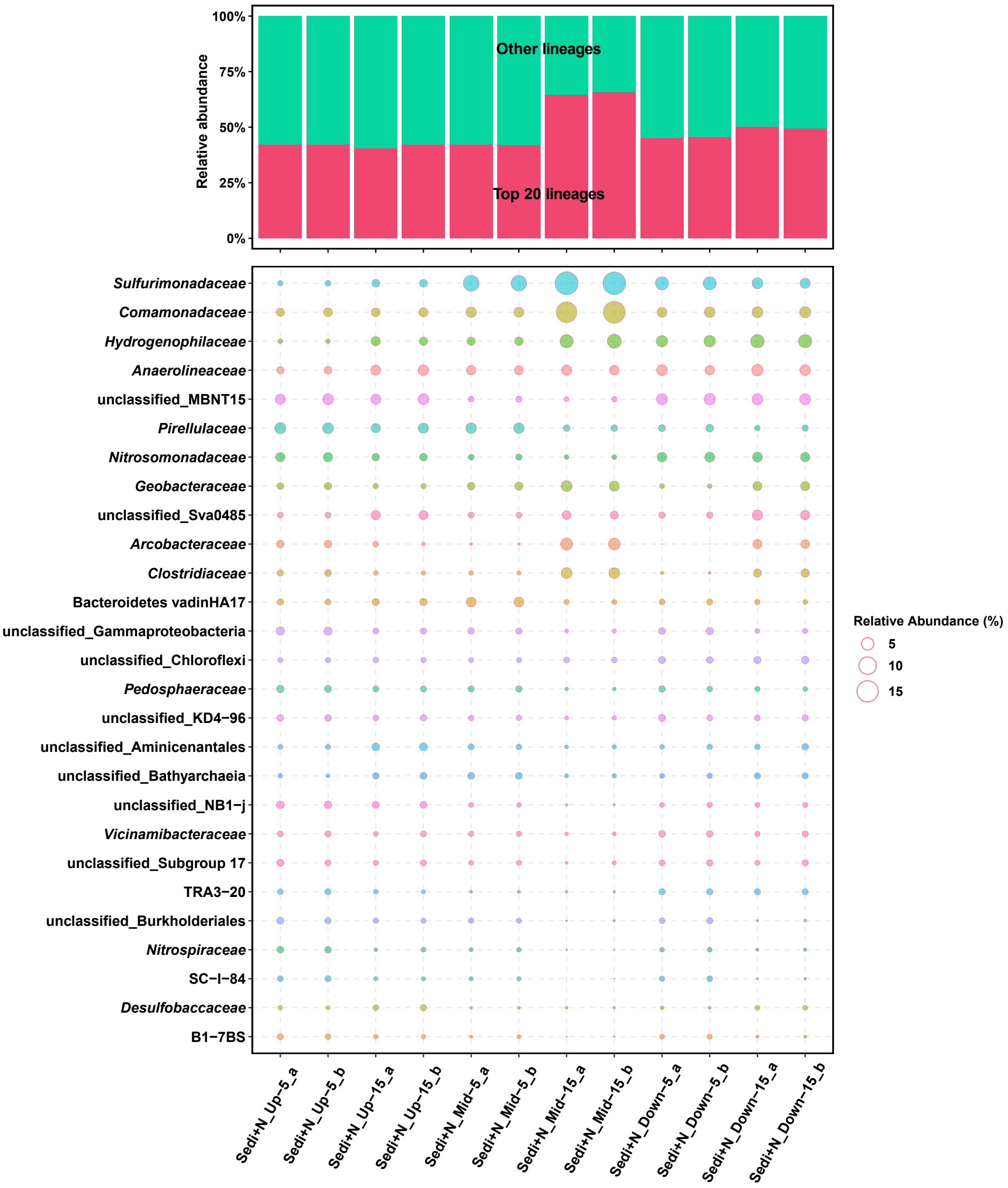

Figure S2: Relative abundance of top 20 most abundant microbial lineages at the family level detected in Schönbrunnen sediments prior to the microcosm incubation (T_0). “Up”, “Mid”, and “Down” in the sample name on the x-axis denote sampling location upstream, midstream, and downstream, respectively. The sample name suffix “a” and “b” suggests biological duplicates. The bar plot on top shows the cumulative abundance of top 20 most abundant family in red color, whereas the green color represents the proportion of other less (OTUs with cumulative relative abundance in all samples less than 7 % were classified within this category) abundant microbial communities at the family level. The bubble plot below shows the relative abundance of each taxon within top 20 most abundant microbial lineages of a sample.

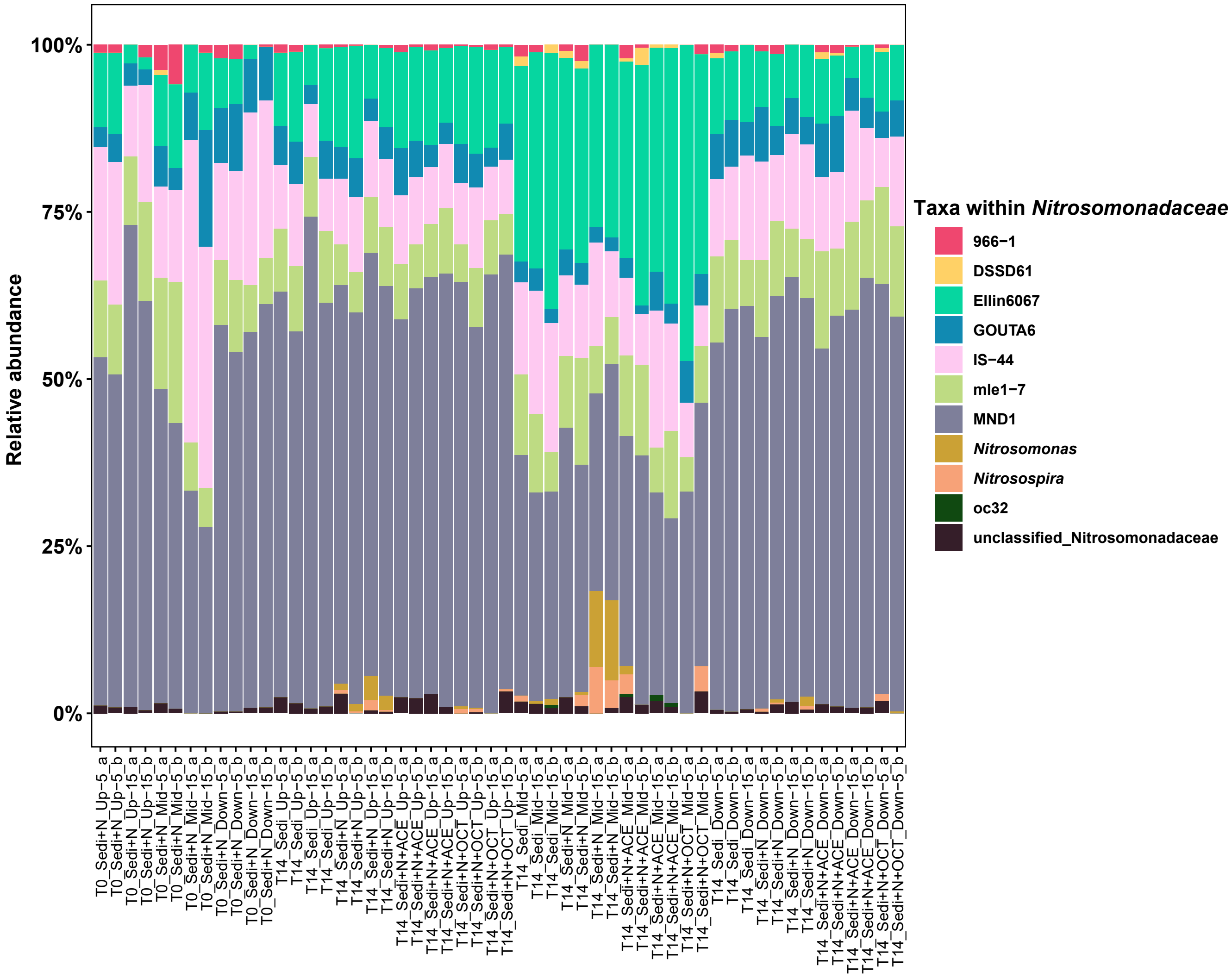

Figure S3: Relative abundance of *Nitrosomonadaceae*-affiliated ASVs clustered at the genus level before and after the microcosm incubation. Plotted genera represent all ASVs detected within the *Nitrosomonadaceae*. T_0 on the x-axis represents day 0 of the microcosm incubation, whereas T_{14} represents the day 14 of the incubation. “Up”, “Mid”, and “Down” on the x-axis indicate sampling location upstream, midstream, and downstream, respectively. “N” stands for the ammonium supplemented incubations. “OCT” indicates 1-octyne treated incubations, whereas “ACE” indicates acetylene treated incubations. The sample name suffixes “a” and “b” indicate biological duplicates.

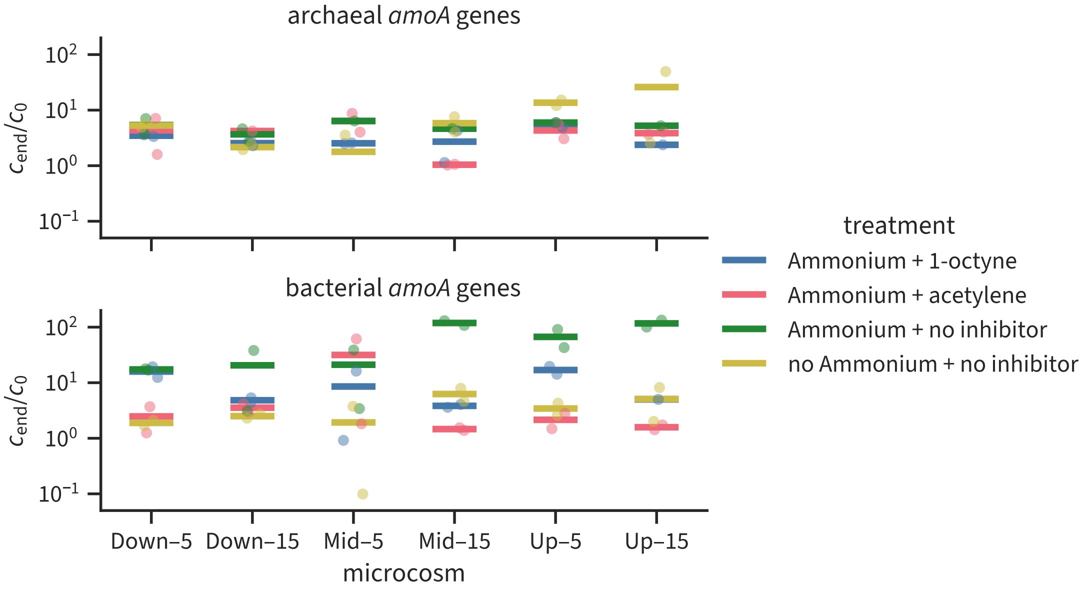

Figure S4: Increase of *amoA* gene concentrations from day 0 to day 14. The bars show the factor by which the concentrations changed for different microcosms and treatments, averaged over three biological replicates. Points indicate individual replicates.

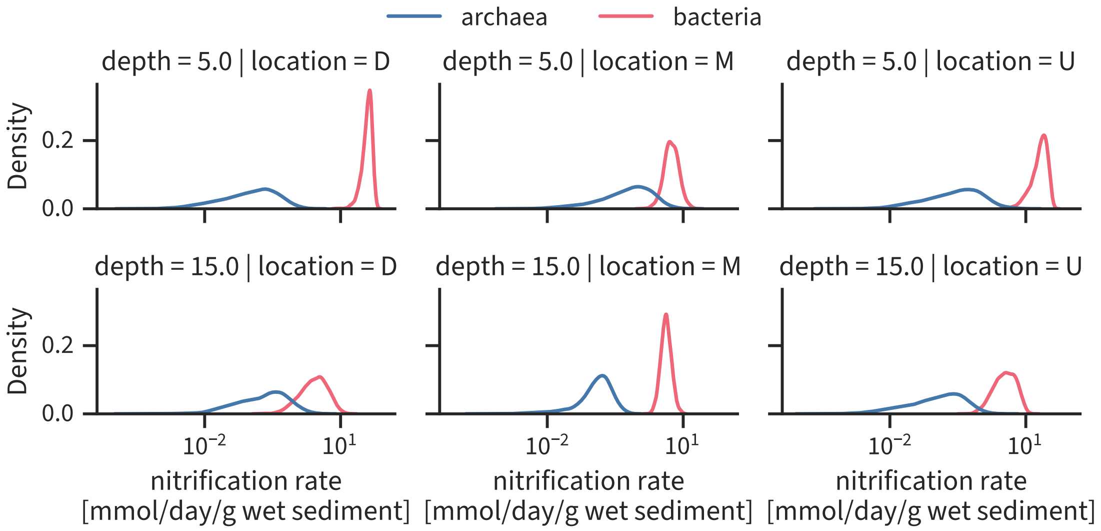

Figure S5: Ammonium oxidation rates calculated with an ammonium concentration of 10 µM, using the *amoA* concentrations measured in the in situ sediment samples, and the posterior samples of kinetic parameters.

4 Bayesian Parameter Estimation for the Reaction Model

4.1 Prior distributions

Prior parameter distributions were based on literature parameter values and physical constraints. For most parameters, a lognormal distribution was chosen for the prior to ensure positivity. In general, broad priors that span several orders of magnitude were chosen. The hyper parameters of the distribution were chosen such that the distribution covers the range of reported parameter values, or that its mode is set to a reported value, when only one value was available. The inhibition factor f_{inhib} ranges between 0 and 1. Therefore, a Beta distribution was chosen for the prior. Since 1-octyne is believed to inhibit ammonia-oxidizing bacteria (AOB) but not ammonia-oxidizing archaea (AOA), the parameters for the Beta distribution were chosen such that the distribution is close to one (no inhibition) for AOA. For AOB, values close to zero (complete inhibition) were favored, but some probability was also given to larger values (up to 60 %, meaning partial inhibition). Table S6 provides an overview of the chosen prior distributions. Table S7 shows the parameters that were not estimated but fixed at literature parameter values, namely the numbers of *amoA* and 16S rRNA genes per cell.

4.2 Preprocessing of measurement data

While *amoA* and 16S rRNA gene data are given in units of genes per mass of wet sediment obtained by centrifuging the slurry in the microcosms, the model computes gene concentrations in units the genes per volume of liquid in the slurry. To compare the simulated with the measured concentrations, both quantities were converted to units of genes copies per volume of slurry. Details of the calculation can be found in a computational notebook published together with the modeling code (Störiko et al., 2025).

For a few individual data points, one out of three biological replicates was excluded for the model calibration because the behavior of the corresponding microcosms deviated strongly from the other two biological replicates. In total, the following points were excluded: 1. Gene data at T14 of replicate 1 in the M5 microcosm because, unlike in the other replicates, they were below the detection limit or an order of magnitude lower than the other replicates. 2. Nitrate data in replicate 1 of the D15 microcosms, because it did not show any nitrification activity. 3. The first replicate of nitrate in M5 at T7, because nitrate dynamics are inconsistent with the other two replicates. While it might be debatable if this data points should be removed, its exclusion did not considerably change the results.

4.3 Sampler convergence

To assess convergence of the sampler, we computed the $\hat{R}$ convergence criterion and the effective sample size (ESS) for all parameters using the ArviZ library (R. Kumar et al., 2019), version 0.17.0. The $\hat{R}$ criterion compares in-chain and between-chain variance of the independent Markov chains, where values far from one indicate non-convergence of the sampler. The $\hat{R}$ criterion was very close to one for nearly all parameters across datasets. Slight convergence issues were only noted for the dataset from M-15, with a maximum $\hat{R}$ of 1.06, and small effective sample sizes (minimum 62) for some parameters. Values for $\hat{R}$ and the effective sample size are tabulated in table S8, which is given in a separate csv file.

4.4 Posterior distributions

Posterior distributions of microbial kinetic parameters for AOA and AOB are shown in fig. S6. Summary statistics for the posterior distributions of other model parameters are tabulated in table S8, which is provided in a separate csv file.

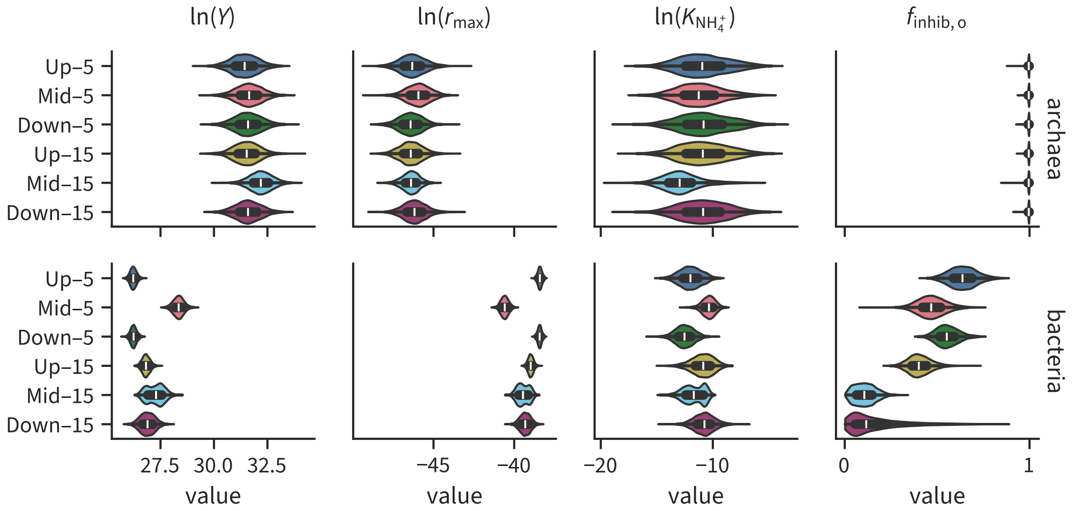

Figure S6: Posterior distributions of model parameters describing the oxidation of ammonium and growth of AOA and AOB for microcosms from different stream segments and depths. Y is the growth yield of ammonium oxidation, r_{max} is the maximum cell-specific reaction rate, $K_{NH_4^+}$ is the half-saturation constant, and f_{inhib} is the inhibition constant of 1-octyne.

Table S6: Model parameters of the reaction model and the corresponding prior distributions for the Bayesian estimation of parameter values.

Parameter	Symbol	Unit	Hyperparameters		Ref.
Parameters with Lognormal distribution[1]			μ	σ	
Maximum NH_4^+ oxidation rate for AOA	$r_{\mathrm{max}}^{\mathrm{AOA}}$	mol cell^{-1} s^{-1}	−44.8	1.0	4
Maximum NH_4^+ oxidation rate for AOB	$r_{\mathrm{max}}^{\mathrm{AOB}}$	mol cell^{-1} s^{-1}	−41.1	1.0	13
Half-saturation constant for AOA	$K_{\mathrm{NH_4^+}}^{\mathrm{AOA}}$	M	−11.5	1.8	3
Half-saturation constant for AOB	$K_{\mathrm{NH_4^+}}^{\mathrm{AOB}}$	M	−7.6	1.5	3
Growth yield for AOA	Y_{AOA}	cells mol^{-1}	32.2	0.5	4
Growth yield for AOB	Y_{AOB}	cells mol^{-1}	28.7	0.5	2
Mixotrophic growth rate constant of AOA	$\mu_{\mathrm{mixotrophic}}^{\mathrm{AOA}}$	s^{-1}	−13.9	1.0	5
Decay constant of AOA and AOB	k_{dec}	s^{-1}	−18.3	0.5	6
NH_4^+ release relative to its oxidation rate	$\alpha_{\mathrm{release}}$	mol mol^{-1}	−2.0	1.0	7
Growth rate of non-nitrifying archaea	r_X^{archaea}	cells L^{-1} s^{-1}	8.1	1.0	8
Growth rate of non-nitrifying bacteria	r_X^{bacteria}	cells L^{-1} s^{-1}	11.1	1.0	8
Nitrogen content of archaeal biomass	β_{archaea}	mol cell^{-1}	−34.5	1.0	9
Nitrogen content of bacterial biomass	$\beta_{\mathrm{bacteria}}$	mol cell^{-1}	−34.5	1.0	9
Initial concentration of AOA in treatment j	$B_{\mathrm{AOA},j}^0$	cells L^{-1}		0.5	10
Initial concentration of AOB in treatment j	$B_{\mathrm{AOB},j}^0$	cells L^{-1}		0.5	10
Initial concentration of archaea in treatment j	$X_{\mathrm{arch},j}^0$	cells L^{-1}		0.5	10
Initial concentration of bacteria in treatment j	$X_{\mathrm{bac},j}^0$	cells L^{-1}		0.5	10
Parameters with Beta distribution[11]			α	β	
Inhibition factor of 1-octyne for AOA	$f_{\mathrm{inhib}}^{\mathrm{AOA}}$	–	100	1.0	12
Inhibition factor of 1-octyne for AOB	$f_{\mathrm{inhib}}^{\mathrm{AOB}}$	–	1	5.0	12

[1] The parameters μ and σ are the mean and standard deviation of the (normally distributed) logarithmic variable, using the natural logarithm. [2] Prosser and Nicol (2012) [3] We chose distributions such that they covered all the values reported in Figure 2c of Jung et al. (2021). [4] Jung et al. (2011)
[5] Tourna et al. (2011) [6] Ding (2010) and Song et al. (2017) [7] The prior was chosen such that the release rate is considerably smaller than ammonium oxidation (centering at about 13 %), since ammonium did not accumulate. [8] The prior was based on a back-of-the-envelope calculation using the data. [9] Based on a biomass composition of $CH_{1.8}O_{0.5}N_{0.2}$ (Cortassa, 2002), and a cell weight of about 10^{-13} g/cell to 10^{-12} g/cell (Loferer-Krößbacher et al., 1998). [10] The mean for initial biomass concentrations is based on the measured initial gene abundances averaged over all treatments and replicates. It varies between stream segments and depths, with the following ranges. AOA: 18.4 to 20.3, AOB: 15.0 to 17.4, archaea: 22.3 to 24.1, bacteria: 23.8 to 24.6. [11] α and β are the shape parameters of the Beta distribution. [12] See section 4.1. [13] Jiang and Bakken (1999)

Table S7: Parameters for the reaction model of the microcosm experiments that were set to fixed values.

Parameter	Value	Unit	Reference
Number of *amoA* copies in AOA	1	genes/cell	Lagostina et al. (2015)
Number of *amoA* copies in AOB	3	genes/cell	Lagostina et al. (2015)
16S rRNA gene copies in AOA	1	genes/cell	Pei et al. (2010)
16S rRNA gene copies in AOB	1	genes/cell	Pei et al. (2010)
16S rRNA gene copies in other archaea	1	genes/cell	Pei et al. (2010)
16S rRNA gene copies in other bacteria	2	genes/cell	Pei et al. (2010)

Table S8: Convergence statistics and summary statistics of the parameter posterior distribution. The $\hat{R}$ convergence criterion and the effective sample size (ESS) provide diagnostics on the sampler convergence. The posterior summary statistics include the median, median absolute deviation (MAD), and the 3rd and 97th percentile. The table is provided in the csv file "table_s8_posterior_statistics.csv".

5 Simulated Concentration Time Series

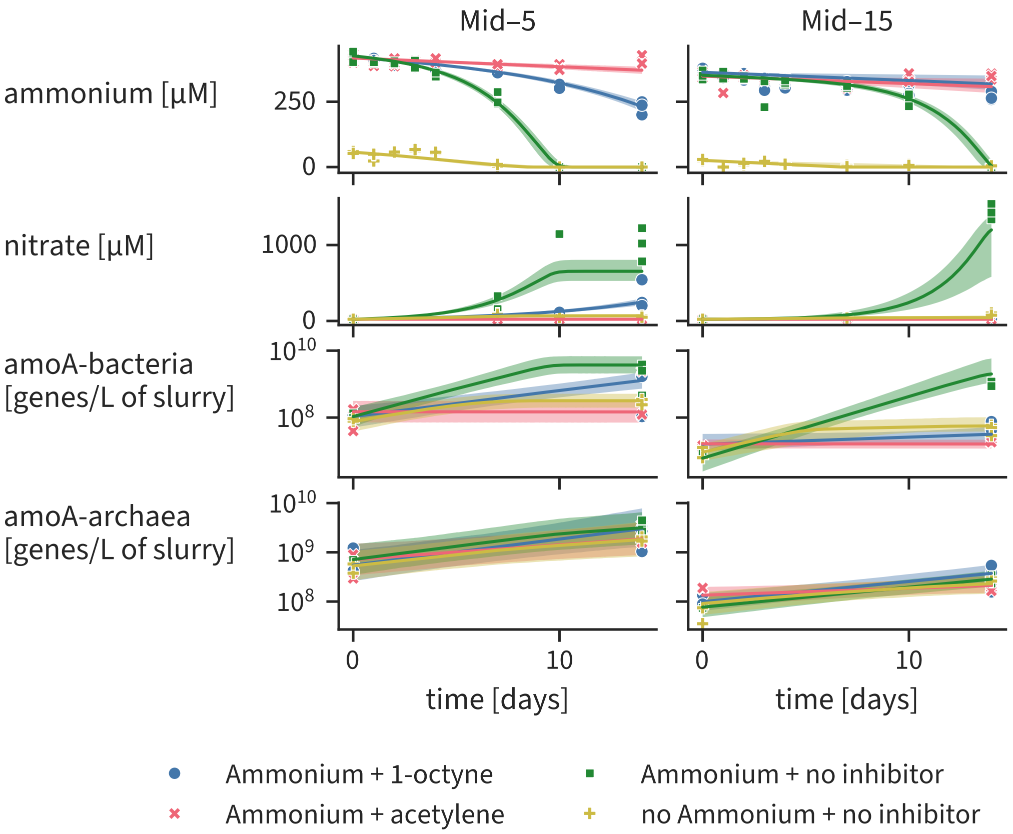

Figure S7: Measured and simulated time series of ammonium, nitrate, and bacterial and archaeal *amoA* gene concentrations for microcosm from midstream 5 cm and 15 cm sediment samples. Shaded areas span between the 10th and 90th percentile, and lines represent the median of the simulated concentrations. Measurements from different biological replicates are indicated by separate markers.

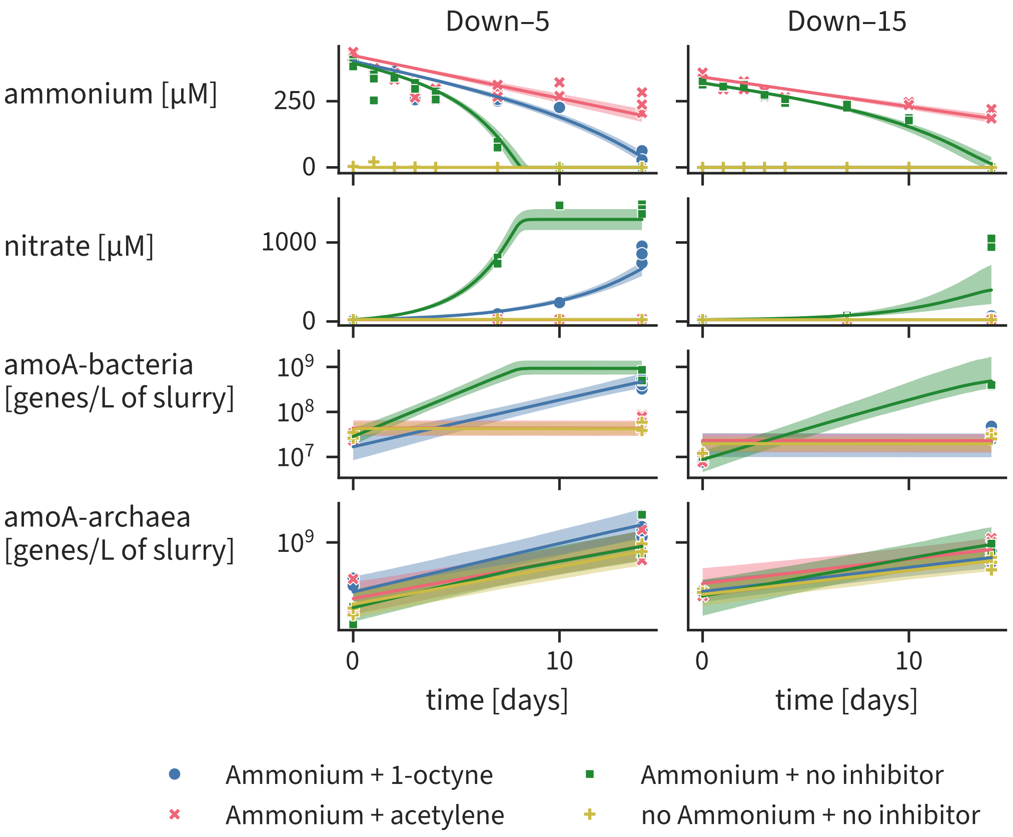

Figure S8: Measured and simulated time series of ammonium, nitrate, and bacterial and archaeal *amoA* gene concentrations for microcosm from downstream 5 cm and 15 cm sediment samples. Shaded areas span between the 10th and 90th percentile, and lines represent the median of the simulated concentrations. Measurements from different biological replicates are indicated by separate markers.

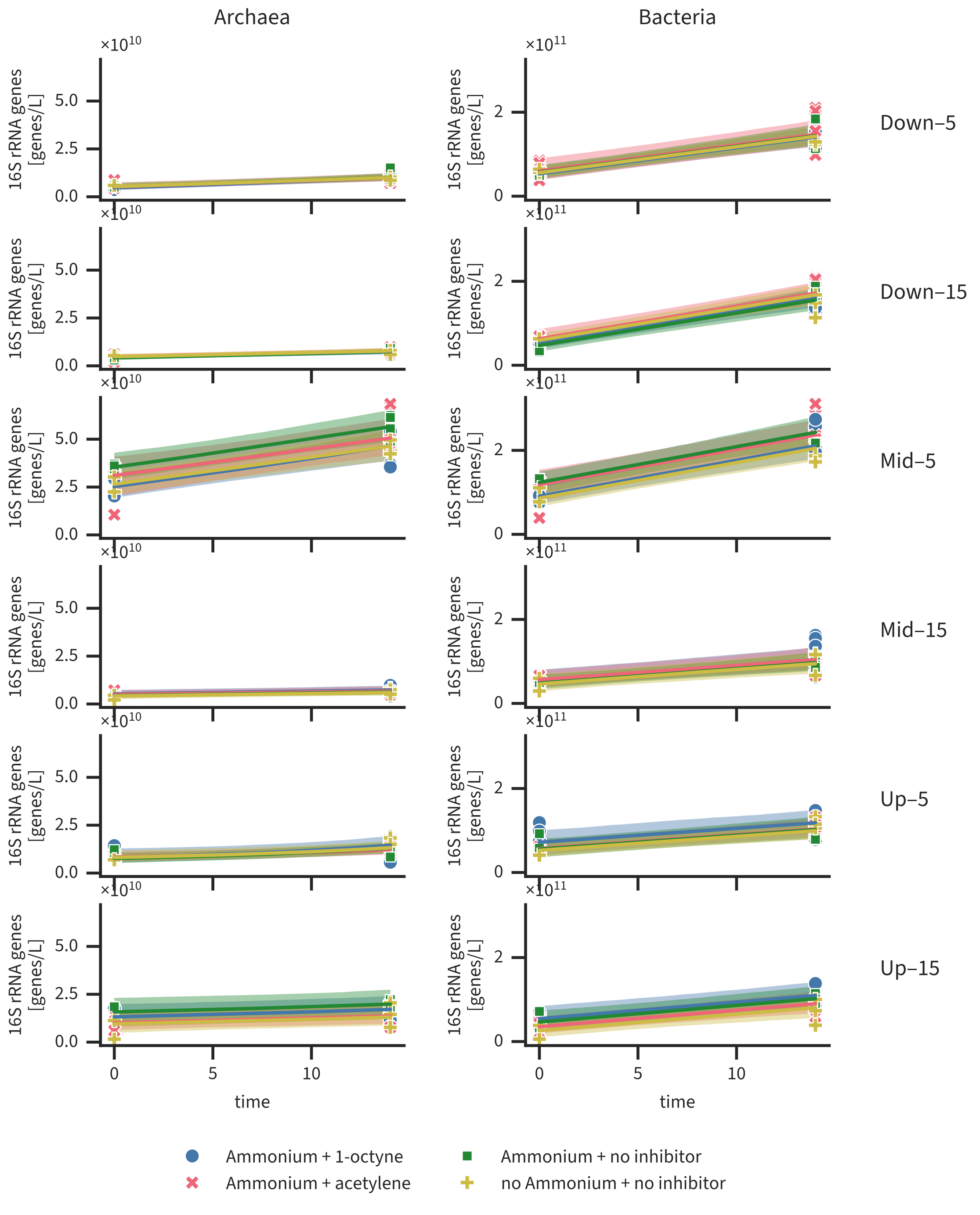

Figure S9: Simulated and measured 16S rRNA gene counts (left: archaea, right: bacteria) for all stream segments and depths.

6 Supplementary References

Altschul, S. F., Gish, W., Miller, W., Myers, E. W., & Lipman, D. J. (1990). Basic local alignment search tool. *Journal of Molecular Biology*, *215*(3), 403–410. https://doi.org/10.1016/S0022-2836(05)80360-2

Apprill, A., McNally, S., Parsons, R., & Weber, L. (2015). Minor revision to V4 region SSU rRNA 806R gene primer greatly increases detection of SAR11 bacterioplankton. *Aquatic Microbial Ecology*, *75*(2), 129–137. https://doi.org/10.3354/ame01753

Benjamini, Y., & Hochberg, Y. (1995). Controlling the false discovery rate: A practical and powerful approach to multiple testing. *Journal of the Royal Statistical Society: Series B (Methodological)*, *57*(1), 289–300. https://doi.org/10.1111/j.2517-6161.1995.tb02031.x

Callahan, B. J., McMurdie, P. J., Rosen, M. J., Han, A. W., Johnson, A. J. A., & Holmes, S. P. (2016). DADA2: High-resolution sample inference from Illumina amplicon data. *Nature Methods*, *13*(7), 581–583. https://doi.org/10.1038/nmeth.3869

Caporaso, J. G., Lauber, C. L., Walters, W. A., Berg-Lyons, D., Lozupone, C. A., Turnbaugh, P. J., Fierer, N., & Knight, R. (2011). Global patterns of 16S rRNA diversity at a depth of millions of sequences per sample. *Proceedings of the National Academy of Sciences of the United States of America*, *108*. https://doi.org/10.1073/pnas.1000080107

Cortassa, S. (2002). *An introduction to metabolic and cellular engineering*. World Scientific.

de la Torre, J. R., Walker, C. B., Ingalls, A. E., Könneke, M., & Stahl, D. A. (2008). Cultivation of a thermophilic ammonia oxidizing archaeon synthesizing crenarchaeol. *Environmental Microbiology*, *10*(3), 810–818. https://doi.org/10.1111/j.1462-2920.2007.01506.x

Di Tommaso, P., Chatzou, M., Floden, E. W., Barja, P. P., Palumbo, E., & Notredame, C. (2017). Nextflow enables reproducible computational workflows. *Nature Biotechnology*, *35*(4), 316–319. https://doi.org/10.1038/nbt.3820

Ding, D. (2010). Transport of bacteria in aquifer sediment: Experiments and modeling. *Hydrogeology Journal*, *18*(3), 669–679. https://doi.org/10.1007/s10040-009-0559-3

Ewels, P. A., Peltzer, A., Fillinger, S., Patel, H., Alneberg, J., Wilm, A., Garcia, M. U., Di Tommaso, P., & Nahnsen, S. (2020). The nf-core framework for community-curated bioinformatics pipelines. *Nature Biotechnology*, *38*(3), 276–278. https://doi.org/10.1038/s41587-020-0439-x

Francis, C. A., Roberts, K. J., Beman, J. M., Santoro, A. E., & Oakley, B. B. (2005). Ubiquity and diversity of ammonia-oxidizing archaea in water columns and sediments of the ocean. *Proceedings of the National Academy of Sciences*, *102*(41), 14683–14688. https://doi.org/10.1073/pnas.0506625102

Jiang, Q. Q., & Bakken, L. R. (1999). Comparison of *Nitrosospira* strains isolated from terrestrial environments. *FEMS Microbiology Ecology, 30*(2), 171–186. https://doi.org/10.1016/S0168-6496(99)00054-9

Jung, M.-Y., Park, S.-J., Min, D., Kim, J.-S., Rijpstra, W. I. C., Damsté, J. S. S., Kim, G.-J., Madsen, E. L., & Rhee, S.-K. (2011). Enrichment and Characterization of an Autotrophic Ammonia-Oxidizing Archaeon of Mesophilic Crenarchaeal Group I.1a from an Agricultural Soil. *Applied and Environmental Microbiology, 77*(24), 8635–8647. https://doi.org/10.1128/AEM.05787-11

Jung, M.-Y., Sedlacek, C. J., Kits, K. D., Mueller, A. J., Rhee, S.-K., Hink, L., Nicol, G. W., Bayer, B., Lehtovirta-Morley, L., Wright, C., de la Torre, J. R., Herbold, C. W., Pjevac, P., Daims, H., & Wagner, M. (2021). Ammonia-oxidizing archaea possess a wide range of cellular ammonia affinities. *The ISME Journal,* 1–12. https://doi.org/10.1038/s41396-021-01064-z

Kumar, R., Carroll, C., Hartikainen, A., & Martin, O. (2019). ArviZ a unified library for exploratory analysis of Bayesian models in Python. *Journal of Open Source Software, 4*(33), 1143. https://doi.org/10.21105/joss.01143

Kumar, S., Stecher, G., Li, M., Knyaz, C., & Tamura, K. (2018). MEGA X: Molecular evolutionary genetics analysis across computing platforms (F. U. Battistuzzi, Ed.). *Molecular Biology and Evolution, 35*(6), 1547–1549. https://doi.org/10.1093/molbev/msy096

Kurtzer, G. M., Sochat, V., & Bauer, M. W. (2017). Singularity: Scientific containers for mobility of compute (A. Gursoy, Ed.). *PLOS ONE, 12*(5), e0177459. https://doi.org/10.1371/journal.pone.0177459

Lagostina, L., Goldhammer, T., Røy, H., Evans, T. W., Lever, M. A., Jørgensen, B. B., Petersen, D. G., Schramm, A., & Schreiber, L. (2015). Ammonia-oxidizing bacteria of the *Nitrosospira* cluster 1 dominate over ammonia-oxidizing archaea in oligotrophic surface sediments near the south atlantic gyre. *Environmental Microbiology Reports, 7*(3), 404–413. https://doi.org/10.1111/1758-2229.12264

Lane, D. J. (1991). 16S/23S rRNA sequencing. *Nucleic acid techniques in bacterial systematics,* 115–175.

Loferer-Krößbacher, M., Klima, J., & Psenner, R. (1998). Determination of bacterial cell dry mass by transmission electron microscopy and densitometric image analysis. *Applied and Environmental Microbiology, 64*(2), 688–694. https://doi.org/10.1128/AEM.64.2.688-694.1998

Love, M. I., Huber, W., & Anders, S. (2014). Moderated estimation of fold change and dispersion for RNA-seq data with DESeq2. *Genome Biology, 15*(12), 550. https://doi.org/10.1186/s13059-014-0550-8

Lueders, T., Pommerenke, B., & Friedrich, M. W. (2004). Stable-isotope probing of microorganisms thriving at thermodynamic limits: Syntrophic propionate oxida-

tion in flooded soil. *Applied and Environmental Microbiology, 70*(10), 5778–5786. https://doi.org/10.1128/AEM.70.10.5778-5786.2004

Martin, M. (2011). Cutadapt removes adapter sequences from high-throughput sequencing reads. *EMBnet.journal, 17*(1), 10. https://doi.org/10.14806/ej.17.1.200

McMurdie, P. J., & Holmes, S. (2013). Phyloseq: An R package for reproducible interactive analysis and graphics of microbiome census data (M. Watson, Ed.). *PLoS ONE, 8*(4), e61217. https://doi.org/10.1371/journal.pone.0061217

McMurdie, P. J., & Holmes, S. (2014). Waste not, want not: Why rarefying microbiome data is inadmissible (A. C. McHardy, Ed.). *PLoS Computational Biology, 10*(4), e1003531. https://doi.org/10.1371/journal.pcbi.1003531

NCBI Resource Coordinators. (2018). Database resources of the National Center for Biotechnology Information. *Nucleic Acids Research, 46*(D1), D8–D13. https://doi.org/10.1093/nar/gkx1095

Oksanen, J., Blanchet, F., Friendly, M., Kindt, R., Legendre, P., Mcglinn, D., Minchin, P., O'hara, R., Simpson, G., Solymos, P., Henry, M., Stevens, H., Szoecs, E., & Maintainer, H. (2019). *Vegan: Community ecology package* (Version 2.5-5). https://CRAN.R-project.org/package=vegan.

Parada, A. E., Needham, D. M., & Fuhrman, J. A. (2016). Every base matters: Assessing small subunit rRNA primers for marine microbiomes with mock communities, time series and global field samples: Primers for marine microbiome studies. *Environmental Microbiology, 18*(5), 1403–1414. https://doi.org/10.1111/1462-2920.13023

Pei, A. Y., Oberdorf, W. E., Nossa, C. W., Agarwal, A., Chokshi, P., Gerz, E. A., Jin, Z., Lee, P., Yang, L., Poles, M., Brown, S. M., Sotero, S., DeSantis, T., Brodie, E., Nelson, K., & Pei, Z. (2010). Diversity of 16S rRNA genes within individual prokaryotic genomes. *Applied and Environmental Microbiology, 76*(12), 3886–3897. https://doi.org/10.1128/AEM.02953-09

Pilloni, G., Granitsiotis, M. S., Engel, M., & Lueders, T. (2012). Testing the limits of 454 pyrotag sequencing: Reproducibility, quantitative assessment and comparison to T-RFLP fingerprinting of aquifer microbes. *PLoS ONE, 7*(7). https://doi.org/10.1371/journal.pone.0040467

Price, M. N., Dehal, P. S., & Arkin, A. P. (2009). FastTree: Computing large minimum evolution trees with profiles instead of a distance matrix. *Molecular Biology and Evolution, 26*(7), 1641–1650. https://doi.org/10.1093/molbev/msp077

Prosser, J. I., & Nicol, G. W. (2012). Archaeal and bacterial ammonia-oxidisers in soil: The quest for niche specialisation and differentiation. *Trends in Microbiology, 20*(11), 523–531. https://doi.org/10.1016/j.tim.2012.08.001

Quast, C., Pruesse, E., Yilmaz, P., Gerken, J., Schweer, T., Yarza, P., Peplies, J., & Glöckner, F. O. (2012). The SILVA ribosomal RNA gene database project: Improved data

processing and web-based tools. *Nucleic Acids Research, 41*(D1), D590–D596. https://doi.org/10.1093/nar/gks1219

R Core Team. (2019). *R: A language and environment for statistical computing*. Vienna, Austria. https://www.R-project.org

Rotthauwe, J. H., Witzel, K. P., & Liesack, W. (1997). The ammonia monooxygenase structural gene *amoA* as a functional marker: Molecular fine-scale analysis of natural ammonia-oxidizing populations. *Applied and Environmental Microbiology, 63*(12). https://doi.org/10.1128/aem.63.12.4704-4712.1997

Song, H.-S., Thomas, D. G., Stegen, J. C., Li, M., Liu, C., Song, X., Chen, X., Fredrickson, J. K., Zachara, J. M., & Scheibe, T. D. (2017). Regulation-structured dynamic metabolic model provides a potential mechanism for delayed enzyme response in denitrification process. *Frontiers in Microbiology, 8*. https://doi.org/10.3389/fmicb.2017.01866

Störiko, A., Wang, Z., Jung, A., Straub, D., Cirpka, O. A., Pagel, H., & Lueders, T. (2025, September 10). *Bayesian simulation of ammonium oxidation in microcosm experiments: Modeling code and data* (Version v2.0.0). Zenodo. https://doi.org/10.5281/zenodo.17087314

Straub, D., Blackwell, N., Langarica-Fuentes, A., Peltzer, A., Nahnsen, S., & Kleindienst, S. (2020). Interpretations of environmental microbial community studies are biased by the selected 16S rRNA (gene) amplicon sequencing pipeline. *Frontiers in Microbiology, 11*, 550420. https://doi.org/10.3389/fmicb.2020.550420

Tourna, M., Stieglmeier, M., Spang, A., Könneke, M., Schintlmeister, A., Urich, T., Engel, M., Schloter, M., Wagner, M., Richter, A., & Schleper, C. (2011). *Nitrososphaera Viennensis*, an ammonia oxidizing archaeon from soil. *Proceedings of the National Academy of Sciences, 108*(20), 8420–8425. https://doi.org/10.1073/pnas.1013488108

Wickham, H. (2016). *Ggplot2: Elegant graphics for data analysis*. Springer-Verlag New York. https://ggplot2.tidyverse.org

Wright, E., S. (2016). Using DECIPHER v2.0 to analyze big biological sequence data in R. *The R Journal, 8*(1), 352. https://doi.org/10.32614/RJ-2016-025
